# Control of aging-associated neurodegeneration via hypothalamic extracellular vesicles containing parathymosin

**DOI:** 10.1016/j.celrep.2025.116561

**Published:** 2025-11-14

**Authors:** Hyun-Gug Jung, Bin Yu, Yuna Choi, Gyeongyun Go, Qichao Zhang, Yizhe Tang, Min Woo Kim, Dongsheng Cai

**Affiliations:** 1 Department of Molecular Pharmacology, Albert Einstein College of Medicine, Bronx, NY 10461, USA; 2 These authors contributed equally; 3 Lead contact

## Abstract

Aging-associated neurodegeneration underlies various neurological diseases; however, the neurocrine basis remains poorly understood. Here, we investigate the role of parathymosin (PTMS), a secretory protein with nuclear functions that has recently been identified as a circulating factor in the brain. The results show that loss of PTMS is sufficient to cause severe, age-dependent neurodegeneration and reduced lifespan, whereas hypothalamic PTMS gain of function counteracts aging-associated brain disorders and extends lifespan. PTMS is present in hypothalamic extracellular vesicles (EVs), particularly in subpopulations released by hypothalamic neural stem/progenitor cells (htNSCs). These htNSC-derived EVs carry small nuclear and nucleolar RNAs in a PTMS-associated manner to protect recipient neurons from DNA damage. Therapeutically, these htNSC-derived EVs provide a strong effect against neurodegenerative disorders associated with PTMS deficiency in mouse models, including Alzheimer’s disease (AD)-like phenotypes in the 5xFAD model. In conclusion, PTMS possesses anti-neurodegenerative properties, and PTMS-containing hypothalamic EVs are significant in combating aging-associated neurodegenerative diseases.

## In brief

Jung, Yu, Choi et al. reveal a critical neuroprotective role of PTMS, while loss of this protein causes severe neurodegeneration. Hypothalamic neural stem cell-derived extracellular vesicles carrying PTMS protect neurons by preventing DNA damage and offer therapeutic benefits against aging-related neurodegenerative and Alzheimer’s-like conditions in animal models.

## Graphical Abstract

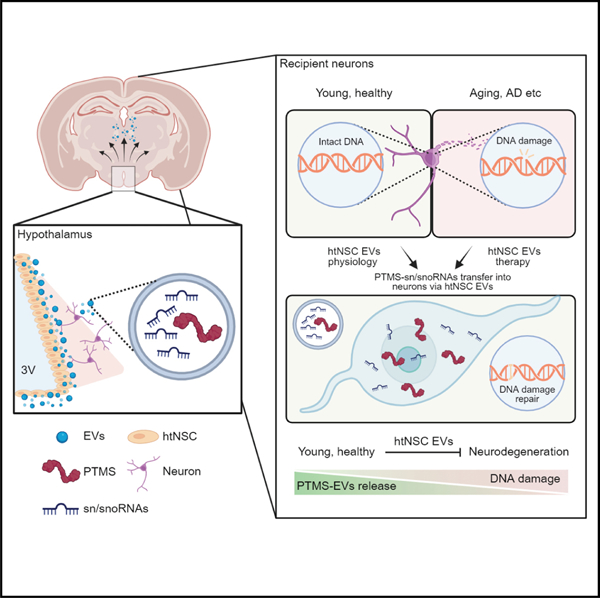

## INTRODUCTION

Aging is not only a key etiological factor that contributes to neuronal loss underlying aging-related neurological disorders but also predisposes genetically susceptible individuals to severe neurodegenerative conditions such as Alzheimer’s disease (AD). However, whether a neurocrine basis exists that links aging to neurodegeneration remains unclear. In the context of our research on the neuroendocrine control of aging by the hypothalamus,^1–4^ we have increasingly focused on the question of how hypothalamic secretory factors influence neurodegeneration. We hypothesize that specific factors released by the hypothalamus play a critical role in protecting against neurodegeneration; however, this protective function diminishes with age due to a decline in the cellular sources for producing these factors, while supplementing these factors may have an anti-neurodegenerative effect. This study aims to rigorously investigate this hypothesis.

Given the regulatory role of hypothalamic neural stem/progenitor cells (htNSCs) during aging,^2^ we have previously conducted a screening for secretory proteins of htNSCs under *in vitro* conditions. From this work, we identified PTMS as a compelling candidate, as it is released by htNSCs and certain neurons, yet is also broadly localized to the nuclei of neurons throughout the brain.^5^ Biochemically, PTMS contains zinc-binding domains and a nuclear localization signal, and it is capable of interacting with histones.^6–8^ To explore its physiological relevance, we generated and studied a genetic mouse model with PTMS loss of function. Notably, this model exhibited severe age-related neurodegeneration, prompting us to further explore the physiological and therapeutic roles of PTMS in aging-associated neurodegeneration, with a particular focus on the neurocrine format of PTMS released by htNSCs and the therapeutic potential. Our findings establish PTMS as a critical anti-neurodegenerative factor and highlight the therapeutic significance of PTMS-containing EVs from htNSCs in combating neurodegenerative disorders such as AD.

## RESULTS

### PTMS genetic loss of function induces severe neurodegeneration in mice

**Figure 1.**
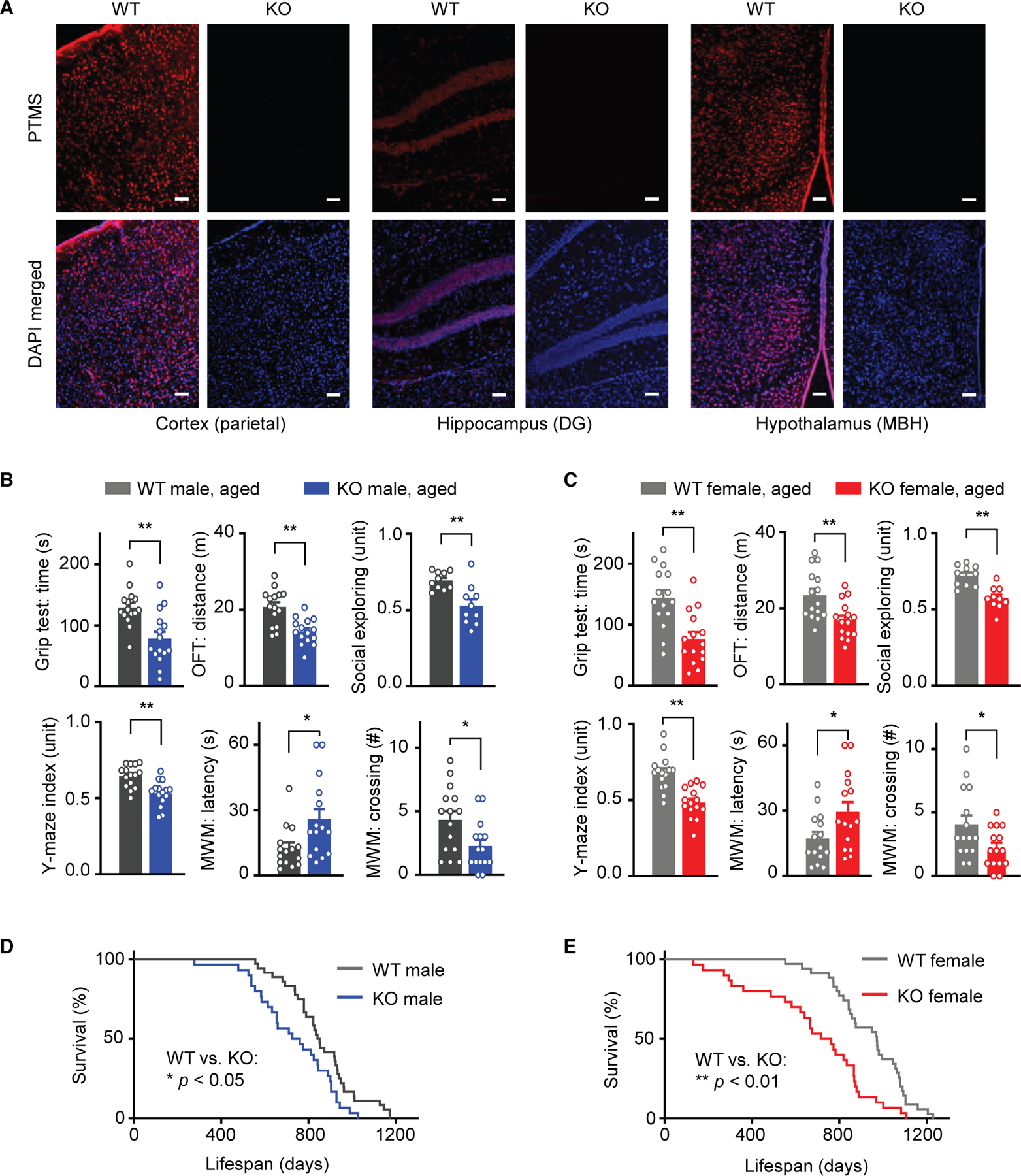
Age-related neurological deficits and reduced lifespan in PTMS-KO mice (A) Representative brain immunostaining from male PTMS-KO mice and littermate WT controls at the age of 3 months. Scale bars, 100 μm. (B and C) Neurobehavioral performance in male (B) and female (C) PTMS-KO and WT mice during aging (21–24 months old). OFT, open field test; MWM, Morris water maze. (D and E) Lifespan of male (D) and female (E) cohorts comparing KO and WT mice. Statistics: *p < 0.05, **p < 0.01, two-tailed unpaired Student’s *t* test (B and C) and log rank (Mantel-Cox) test (D and E); *n* = 10–15 mice per group (B and C), and *n* = 30–36 mice per group (D and E); data in bar graphs are presented as mean ± SEM. See Figures S2 and S3 for additional information.

**Figure 2.**
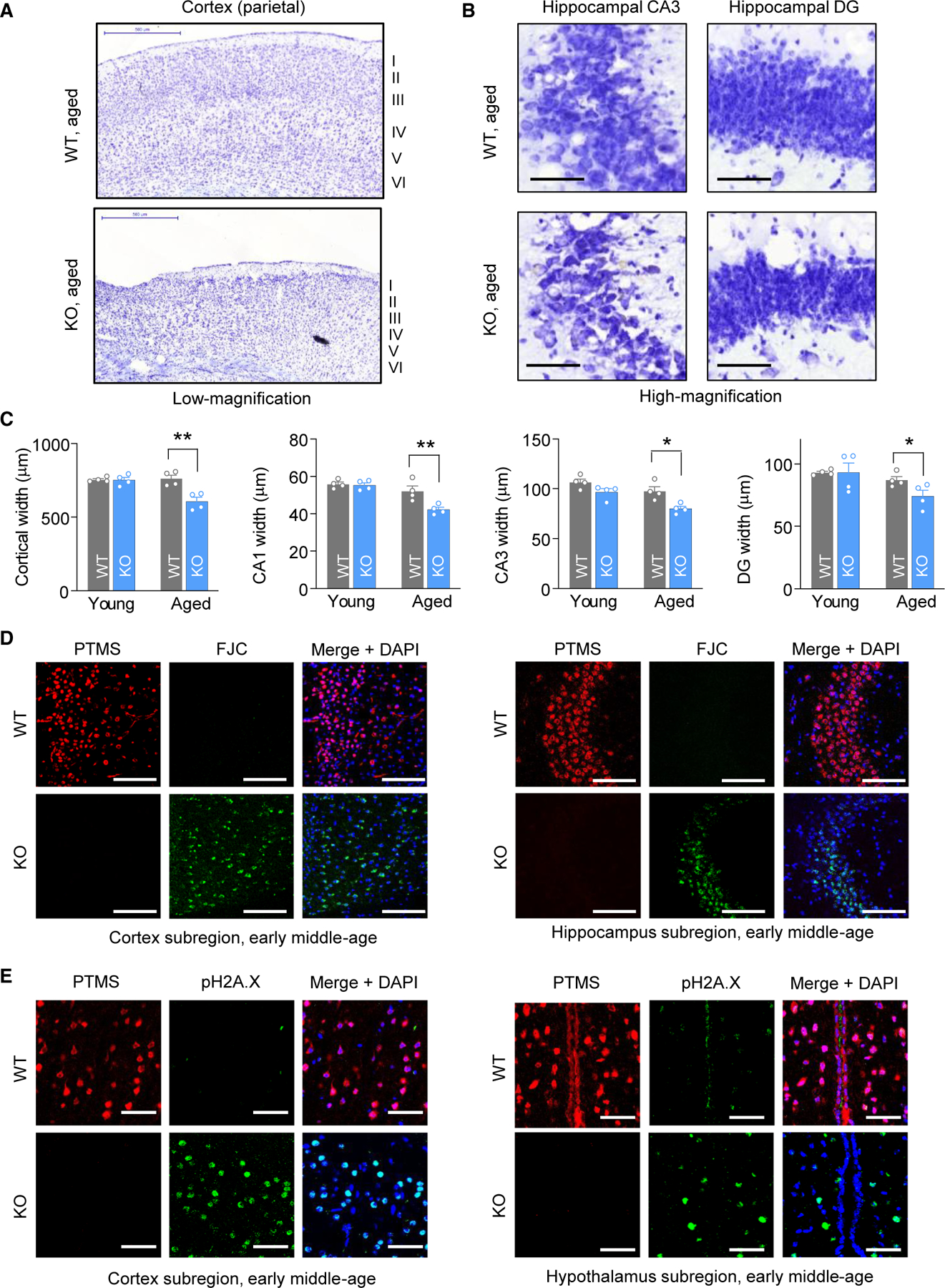
Age-related neurodegeneration and DNA damage in PTMS-KO mice (A–C) Nissl staining of brain sections from aged (24-month-old) male PTMS-KO and littermate wild-type (WT) mice. (A) Representative low-magnification images of the parietal cortex. (B) High-magnification images of hippocampal subregions. (C) Quantification for the thicknesses of the parietal cortex and hippocampal subregions in both young (4 months old) and aged (24 months old) PTMS-KO and WT mice. Scale bars, 0.5 mm (A) and 25 μm (B). Statistics: *p < 0.05, **p < 0.01, two-way ANOVA with Tukey’s post hoc test; *n* = 4 mice per group; data are presented as mean ± SEM. (D and E) Brain sections from male PTMS-KO and WT littermate controls at early middle age (10 months old) were analyzed by Fluoro-Jade C (FJC) staining (D) and phosphorylated H2A.X (pH2A.X) immunostaining (E). Images show the subregions of anterior cingulate cortex, hippocampal CA3 curvature, and mediobasal hypothalamus. Scale bars, 100 (D) and 50 μm (E). See Figures S4 and S5 for additional information.

To investigate the physiological role of PTMS *in vivo*, we employed a genetic strategy to develop a PTMS loss-of-function mouse model. Specifically, we generated a PTMS knockout (KO) mouse model by deleting the genomic region spanning exons 2 through 5, thereby removing the majority of the coding sequence (Figure S1). The KO model was established on a C57BL/6 background. Heterozygous progenies were inter-crossed to generate homozygous KO mice and wild-type (WT) littermate controls. Loss of PTMS expression in the brains of KO mice was confirmed by immunostaining (Figure 1A) and also by other approaches such as western blot and qPCR (data not shown). Young PTMS-KO mice appeared to be normal, exhibiting typical development and growth, and performed similarly when compared to WT controls across a panel of neurobehavioral assays (Figure S2). In contrast, as the mice aged, KO mice performed worse than WT controls in these neurobehavioral assays, with deficits becoming pronounced during aging (Figures 1B, 1C, and S3). Meanwhile, we established a large cohort of PTMS-KO and WT mice for lifespan follow-up. Longitudinal monitoring revealed that KO mice exhibited a significantly shortened lifespan compared to WT controls (Figures 1D and 1E). To further investigate age-related changes, we examined brain histology in a group of young versus aged PTMS-KO and WT mice. While no differences were observed in gross brain morphology at a young age (Figure S4), aged PTMS-KO mice exhibited pronounced brain atrophy affecting multiple regions (Figures 2A–2C). In this context, we investigated whether the observed aging-associated brain atrophy was related to earlier neurodegeneration by employing two methods: Fluoro-Jade C staining, a chemical marker of neurodegeneration, and phosphorylated H2A.X (pH2A.X) immunostaining, a biomarker of DNA damage. The results revealed clear manifestations of neurodegeneration in representative brain regions of animals during early middle age, as evidenced by Fluoro-Jade C staining (Figures 2D and S5A) and pH2A.X immunostaining (Figures 2E and S5B). Together, these findings demonstrate that PTMS deficiency leads to progressive neurodegeneration and functional neurological decline in mice.

### Protection against aging-associated neurodegeneration by hypothalamic PTMS

**Figure 3.**
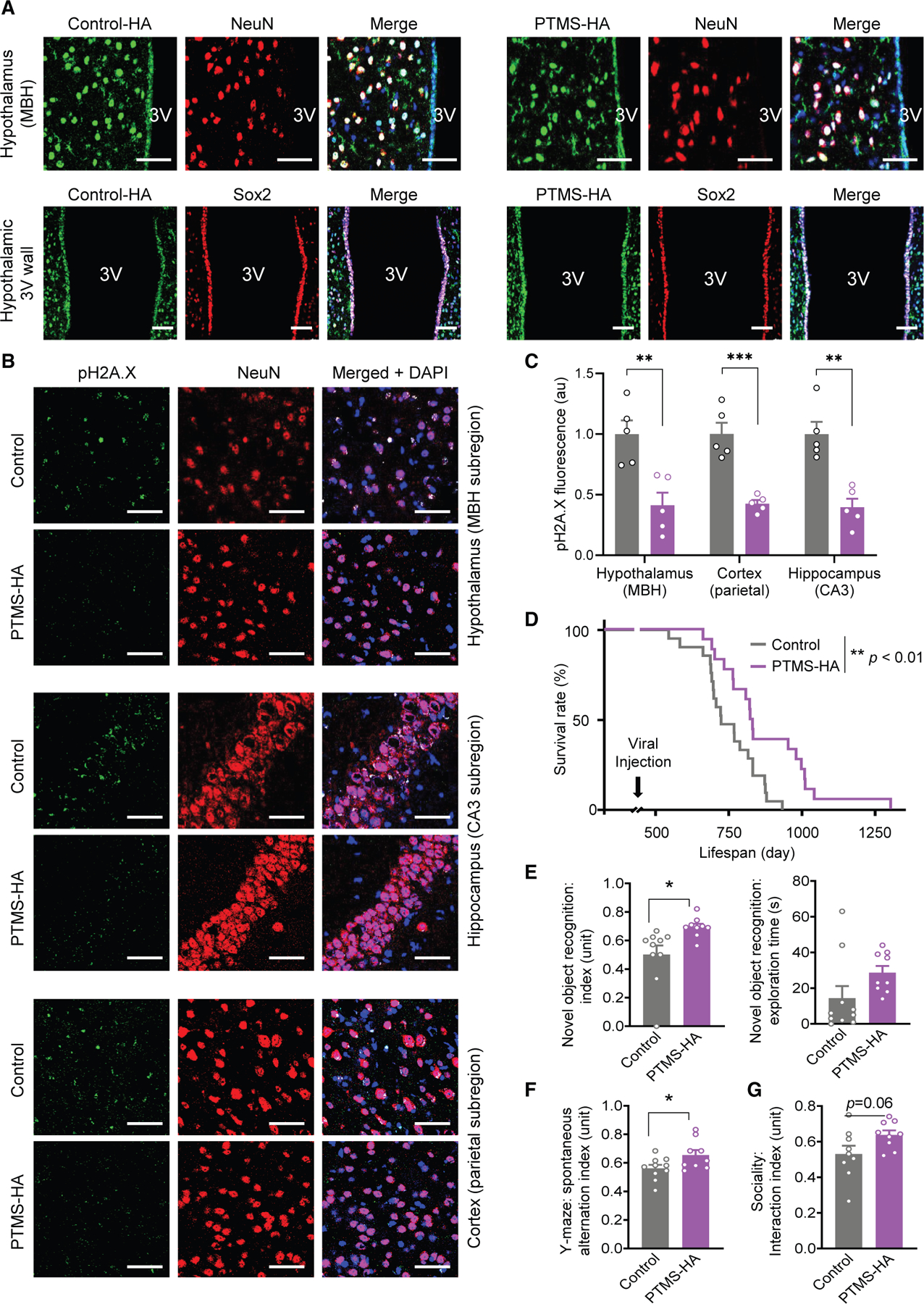
Neuroprotective effects by hypothalamic PTMS gain of function (A) Hypothalamic PTMS gain-of-function model was established by stereotactic delivery of lentiviruses encoding HA-tagged PTMS. Lentiviruses under a CMV promoter were injected into the 3V (targeting htNSCs along the 3V wall), and lentiviruses under a synapsin promoter were bilaterally injected into the MBH to target local neurons. Matched controls received lentiviruses encoding HA-tagged scramble sequence. Immunostaining confirmed transduction of both htNSCs (Sox2-positive) and neurons (NeuN-positive) in the MBH. Scale bars, 50 μm. (B and C) A group of hypothalamic PTMS gain-of-function models and controls was generated using standard C57BL/6 mice (∼15 months old, male) and maintained 1 year before examination for aging-associated neuronal DNA damage via brain immunostaining for phosphorylated H2A.X (pH2A.X) (B) and quantitative analysis (C). (D) Lifespan following PTMS gain of function in standard male C57BL/6 mice since middle age (∼15 months old), compared to matched control group. (E–G) A group of PTMS gain of function and control were generated using C57BL/6 mice (∼15 months old, male) and maintained 6 months before neurobehavioral assays (one death occurred in control group prior to sociality test). Scale bars, 50 μm. Statistics: *p < 0.05, **p < 0.01, ***p < 0.001, two-tailed unpaired Student’s *t* test (C and E–G) and log rank (Mantel-Cox) test (D); *n* = 5 mice (C), *n* = 18–21 mice (D), and *n* = 9–10 mice per group (E–G); data in bar graphs are presented as mean ± SEM. See Figure S6 for additional information.

In parallel with the loss-of-function studies in Figures 1 and 2, we developed a gain-of-function approach targeting the hypothalamus to test whether increasing hypothalamic PTMS levels could mitigate aging-related neurodegeneration. Our target was focused on the mediobasal hypothalamus (MBH), the location containing htNSCs that we previously identified as a key source of releasing PTMS.^5^ Using stereotactic delivery, we introduced a recombinant lentivirus encoding hemagglutinin (HA)-tagged PTMS into the MBH of middle-aged mice. The lentiviruses were delivered locally into the third ventricle (3V) to target the htNSC population, also known as Sox2-positive tanycytes lining the 3V wall. To expand the MBH coverage and given that neurons in this region are also significant in releasing PTMS,^5^ we additionally included bilateral injections of lentiviral PTMS under a neuron-specific promoter directly into the MBH parenchyma, thereby increasing the neuronal expression of PTMS in this region. Successful target of both htNSCs and MBH neurons was confirmed by immunostaining for HA-tagged PTMS versus HA-tagged scramble controls (Figure 3A). Low-magnification hypothalamic imaging, along with examination of extra-hypothalamic regions, confirmed the success of MBH-targeted lentiviral delivery (Figure S6). A cohort of PTMS gain-of-function and control mice was thus established at middle age and monitored longitudinally into aged conditions. As shown in Figures 3B and 3C, aged control mice exhibited extensive neuronal DNA damage across multiple brain regions, whereas mice with PTMS gain of function displayed a significant reduction in these pathologies, suggesting that hypothalamic PTMS is anti-neurodegenerative. To assess whether this neuroprotection translated into physiological benefits, we conducted a lifespan study in a larger cohort. Remarkably, hypothalamic PTMS gain of function conferred a significant longevity advantage (Figure 3D). Consistently, neurobehavioral assay on a group of this model revealed that mice with hypothalamic PTMS gain of unction outperformed the controls (Figures 3E–3G). Together, these results demonstrate that increasing hypothalamic PTMS levels can attenuate aging-related neurodegeneration, enhance cognitive function, and extend lifespan.

### Subtypes of hypothalamic EVs are characterized by the presence of PTMS

**Figure 4.**
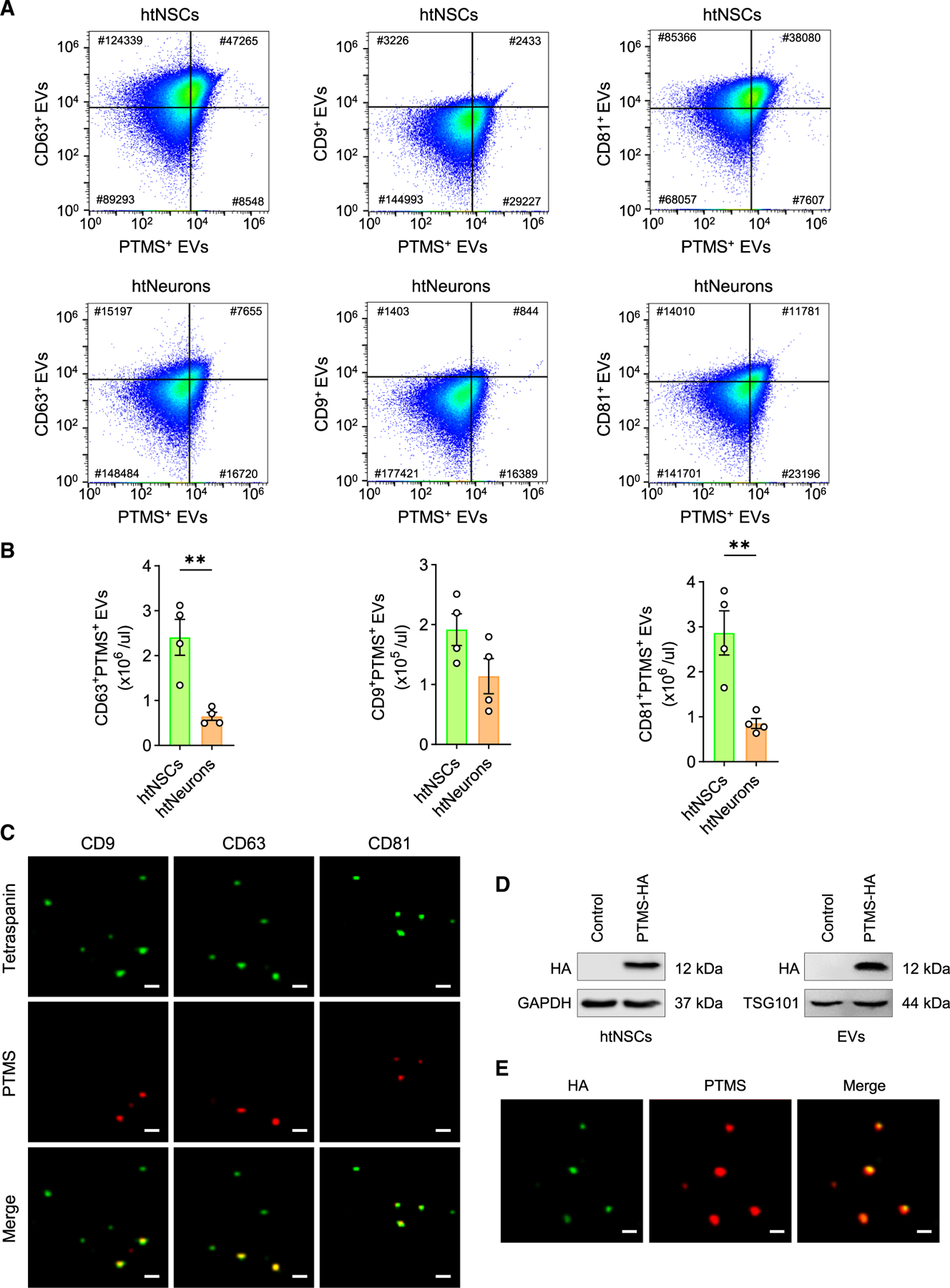
The presence of PTMS in htNSC-EV subpopulations (A and B) Using *in vitro* models of htNSCs versus hypothalamic neurons (htNeurons) based on mHypoA-2/21, EVs collected from these cells over the same cell number and same time were analyzed by nano-flow cytometry (FCM) for PTMS and canonical EV tetraspanins (CD9, CD63, CD81). (A) Representative compensated FCM plots; (B) quantitative analysis showing the relative abundance of PTMS-positive EVs within each tetraspanin-defined subpopulation. Statistics: **p < 0.01, two-tailed unpaired Student’s *t* test; *n* = 4 independent biological replicates per group; data are presented as mean ± SEM. (C) Purified EVs from htNSCs were subjected to co-immunostaining for PTMS and tetraspanins CD9, CD63, and CD81. Scale bars, 1 μm. (D and E) htNSCs transduced with lentiviral HA-tagged PTMS were analyzed for HA expression by western blot assay at the cellular and EV levels (D) and by co-immunostaining to visualize HA incorporation into EVs (E). Scale bars, 1 μm. See Figure S7 for additional information.

While the hypothalamus is classically recognized for secreting neuropeptides, we previously uncovered an additional role for htNSCs in releasing EVs.^2^ This context prompted us to investigate whether htNSCs might function as a source of PTMS-containing EVs, a plausible hypothesis given that PTMS is an acidic protein and likely requires a carrier for long-distance circulation. To address this question, we utilized a purified *in vitro* htNSC model developed in our prior studies.^2,9–12^ These htNSCs were enriched through serum-free neurosphere culture in N2/B27 medium, which supports self-renewal of htNSCs while depleting other cell types. After the process of serial passaging in the form of neurospheres, the resulting cell population was enriched for htNSCs. For comparison, we included a hypothalamic neuronal cell line (htNeuron) based on the mHypoA-2/21 cell model, which has previously been validated in research.^13,14^ Equal numbers of htNSCs and htNeurons were cultured in EV-depleted medium, and EVs released over the same time period were collected and purified by ultracentrifugation. EVs were analyzed using nano-flow cytometry for canonical tetraspanin markers (CD9, CD63, CD81), together with PTMS detection to define PTMS-positive populations. Both cell types were found to secrete PTMS-positive EVs; however, htNSCs released them at significantly higher levels (Figures 4A and 4B). Immunofluorescence staining further confirmed the presence of endogenous PTMS within a subpopulation of htNSC-derived EVs across all three tetraspanin-defined EV subtypes (Figure 4C). To further assess the loading of PTMS into EVs, we transduced htNSCs with a lentiviral vector encoding HA-tagged PTMS (Figure 4D). Immunostaining for the HA epitope indicated efficient incorporation of PTMS into htNSC-EVs (Figures 4E and S7). Taken together, these findings demonstrate that htNSCs are a significant source of PTMS-positive EVs in the hypothalamus and are capable of producing both endogenous and engineered forms of this neuroprotective cargo.

### PTMS associates with small nuclear and nucleolar RNAs in htNSC-derived EVs

**Figure 5.**
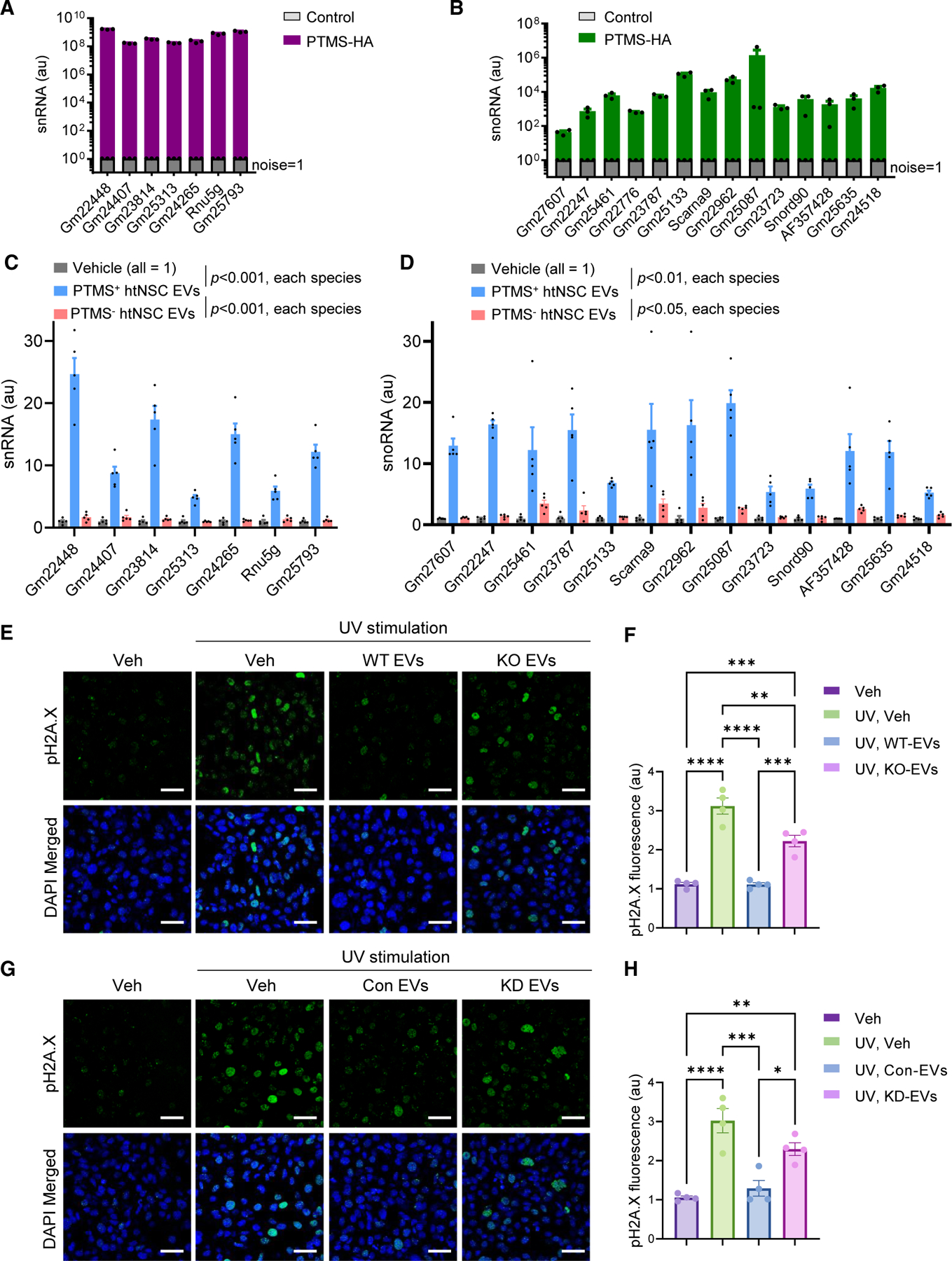
PTMS-sn/snoRNA association in htNSC-EVs and role against DNA damage (A and B) EVs purified from htNSCs expressing HA-tagged PTMS were subjected to HA immunoprecipitation, followed by qPCR to detect associated sn/snoRNA species. EVs from cells expressing HA-tagged control construct were used as negative controls. (C and D) Hypothalamic neuronal GT1–7 cells were pretreated with actinomycin D (2.5 μg/mL, 24 h) to block *de novo* transcription, followed by 1 h treatment with 5 μg/mL of purified EVs from two htNSC versions, established from newborn WT mice (labeled as WT EVs) and PTMS-KO mice (labeled as KO EVs), respectively. Vehicle treatment was included for basal control. Cells were then thoroughly washed and collected for qPCR measurement of candidate sn/snoRNAs. Vehicle treatment was used as baseline control. (E and F) GT1–7 cells were pretreated with WT EVs vs. KO EVs (5 μg/mL) or vehicle for 1 h, then exposed to UV irradiation for 2 h to induce nuclear DNA damage, followed by immunostaining for phosphorylated H2A.X (pH2A.X). Vehicle treatment without UV exposure was included as the baseline control. (G and H) GT1–7 cells were treated with 5 μg/mL EVs derived from htNSCs with Dicer knockdown (KD) vs. matched control (labeled as KD EVs and Con EVs, respectively) or vehicle, then subjected to 2 h UV exposure before being processed for pH2A.X immunostaining. Vehicle treatment without UV exposure was included as the baseline control. Scale bars, 50 μm. Statistics: *p < 0.05, **p < 0.01, ***p < 0.001, ****p < 10^−4^, two-tailed unpaired Student’s *t* test (A and B), and one-way ANOVA with Tukey’s post hoc test (C, D, F, and H); *n* = 3 independent biological replicates (A and B), and *n* = 4–5 independent biological replicates (C, D, F, and H); data are presented as mean ± SEM. See Figures S8 and S9 for additional information.

Then, we focused on studying htNSC-derived EVs to profile the molecular cargos, which could be associated with PTMS. Because htNSC-derived EVs are rich with diverse small RNA species,^2,9,10,15^ and given the nuclear localization of PTMS in the brain,^5^ we hypothesized that PTMS may have an association with nucleus-related small RNAs, such as small nuclear RNAs (snRNAs) and small nucleolar RNAs (snoRNAs). To explore this possibility, we studied a line of htNSCs transduced with a lentiviral construct encoding HA-tagged PTMS (Figure 4D), which provided a system to perform HA-based immunoprecipitation (IP) assays for total proteins of the EVs released by these cells. We compiled a panel of approximately 50 sn/snoRNAs based on recent literature^16^ and screened for expression in our htNSC model using qPCR with species-specific primers. Approximately 20 sn/snoRNAs were confirmed to be evidently expressed in htNSCs and were therefore selected for further IP analysis. HA-IP was performed on the protein lysates of purified htNSC-derived EVs, and the specificity was validated by using immunoglobulin G (IgG) as an isotype control. Our qPCR profiling revealed that multiple sn/snoRNA species were contained in the HA-PTMS pull-down (Figures 5A and 5B). The same HA-IP procedure was performed using EVs derived from a non-hypothalamic neuronal cell line (Neuro-2A), which did not clearly yield detectable pull-down of these sn/snoRNAs (Figure S8). While a systematic examination of other neuronal and neural cell models is beyond this study, our findings suggest that the PTMS-sn/snoRNA complex constitutes a cargo type in htNSC-derived EVs, a feature that might be absent or less present in EVs from other neural cell types. Moreover, given that both PTMS and RNA molecules are acidic, it is plausible that one or more RNA-binding proteins may be involved in facilitating the formation or stabilization of PTMS-small RNA complexes, an open question for future investigation. Nonetheless, our results indicate that PTMS is associated with small RNAs, particularly snRNAs and snoRNAs, within htNSC-derived EV subpopulations.

### PTMS-associated small RNAs are transferred into neurons via htNSC-derived EVs

Next, we investigated whether PTMS-associated sn/snoRNAs could be transferred to recipient cells via PTMS-containing htNSC-derived EVs. Based on prior studies indicating efficient uptake of htNSC-derived EVs by neurons, we used the GT1–7 neuronal cell line as a recipient model. To better isolate transferred RNA species, recipient cells were pretreated with a transcriptional inhibitor to block endogenous RNA synthesis. To minimize secondary effects, GT1–7 cells were exposed to htNSC-EVs only briefly for 1 h, and subsequently cells were thoroughly washed and harvested for candidate small RNA assay via qPCR. To assess PTMS-dependent delivery, we compared EVs that were released by cultured htNSCs obtained from WT versus PTMS-KO mice. These EVs exhibited comparable size profiles and expression levels of canonical tetraspanins (Figure S9), suggesting that the loss of PTMS did not markedly alter the general characteristics of these EVs. The results demonstrated that PTMS-positive EVs delivered sn/snoRNAs into recipient cells, as evidenced by an evident increase in these RNA species following the EV exposure (Figures 5C and 5D). In contrast, cells treated with PTMS-missing EVs did not lead to such an increase. These observations indicate that PTMS is essential for associating with sn/snoRNAs in htNSC-EVs and for facilitating the transfer of this complex into recipient neurons. All together, these findings demonstrate that PTMS as a molecular partner of sn/snoRNAs within certain hypothalamic EVs, enabling sn/snoRNA delivery to target cells.

### Protection against DNA damage by htNSC-derived EVs containing PTMS

Recent studies have implicated sn/snoRNAs in cellular defense mechanisms against DNA damage.^17–20^ Supported by our finding that PTMS facilitates the transfer of sn/snoRNAs via htNSC-EVs, we investigated whether this PTMS-sn/snoRNA complex might contribute to genomic protection in recipient neurons. To address this question, we continued to employ the *in vitro* model of the GT1–7 neuronal cell line by exposing a dose of ultraviolet (UV) light to cause nuclear DNA damage in these cells. We compared the effects of PTMS-containing versus PTMS-missing htNSC EVs, which were described in Figures 5C, 5D, S9A, and S9B. Following UV exposure, recipient cells displayed elevated levels of pH2A.X, a canonical marker of DNA damage. UV-induced pH2A.X was attenuated in these cells when treated with PTMS-containing htNSC EVs, but much less with PTMS-missing EVs (Figures 5E and 5F), indicating that PTMS confers protection against neuronal DNA damage. To further test whether small RNAs could be accountable for this effect, we generated a Dicer knockdown model in htNSCs to impair biogenesis of small RNAs. Dicer knockdown led to an about 70% reduction in small RNA content within htNSC-EVs (Figures S9C and S9D), while overall profiles such as EV size distribution and tetraspanin expression were not evidently altered. We found that Dicer-deficient htNSC-derived EVs failed to protect recipient neurons from UV-induced pH2A.X accumulation (Figures 5G and 5H), mirroring the effects of PTMS-missing EVs. Taken together, these results demonstrate that the PTMS-small RNA complex within htNSC-derived EVs plays a significant role in protecting recipient neurons from DNA damage. This EV-mediated neuroprotection may provide a therapeutic basis for mitigating neurodegeneration associated with genomic instability.

### Partial reversal of neurological deficits in the PTMS knockout model by htNSC-EVs

**Figure 6.**
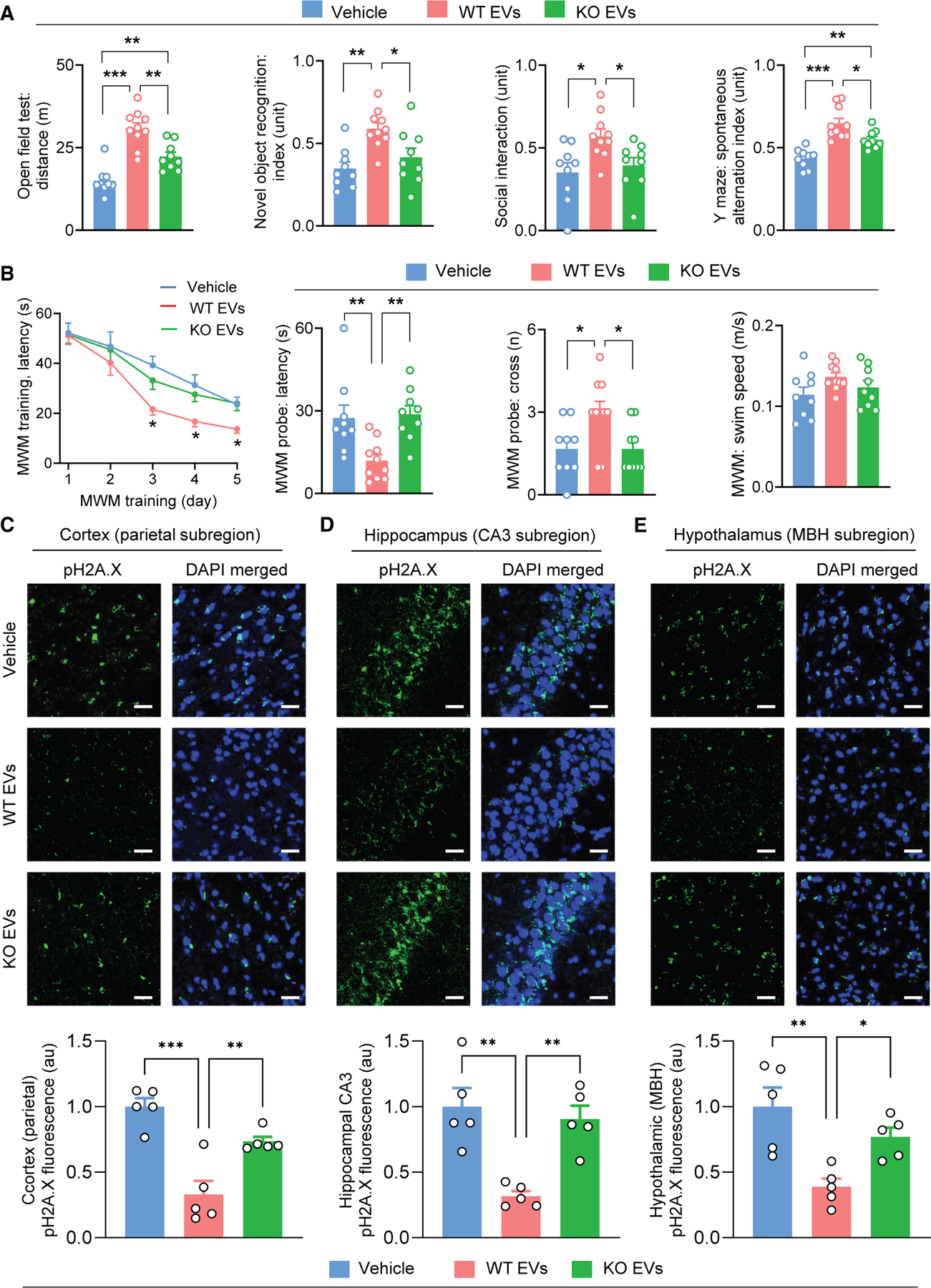
Partial reversal of neurological deficits in the PTMS-KO model by htNSC-EVs Middle-aged PTMS-KO mice (15 months old, male) were i.c.v. administrated via pre-implanted cannulas in the 3V with 100 ng purified EVs from two htNSC versions, established from newborn WT mice (labeled as WT EVs) and PTMS-KO mice (labeled as KO EVs), respectively. Vehicle treatment was included for basal control. Treatments were administered three times per week for 3 months. After treatment, mice were evaluated for neurobehavioral performance using open field test, novel object recognition (NOR), social interaction, and Y maze (A), followed by Morris water maze (MWM) assessment (B). Finally, subsets of mice were used for brain immunostaining per DNA damage marker pH2A.X, followed by quantification (C–E). Scale bars, 25 μm. Statistics: *p < 0.05, **p < 0.01, ***p < 0.001, one-way ANOVA with Tukey’s post hoc test; *n* = 9–10 mice per group (A and B), and *n* = 5 mice per group (C–E). Statistical *p* values in the curve graph (B, left) represent comparations between WT EVs and each of the other two groups at the matched time points. Data are presented as mean ± SEM. See Figures S10 and S11 for additional information.

We then decided to study whether the neurological deficits in PTMS-KO mice could be ameliorated by htNSC-derived EVs and if PTMS is an important cargo. Prior to therapeutic intervention, we confirmed neuronal uptake of htNSC-derived EVs in multiple brain regions following an intracerebroventricular (ICV) administration at a therapeutic dose. EVs were labeled with the fluorescent dye PKH26 for brain tracking. The specificity of this method was demonstrated by clear fluorescence in the brain after PKH26-EV injection, whereas vehicle injection did not lead to such a fluorescent signal (Figure S10). Neuronal immunostaining was applied to brain sections to assess if neurons represented a key recipient of these EVs. Although technically PKH26 fluorescence was dampened due to immunostaining procedure, it was still evident that these EVs were taken predominantly by neurons across various brain regions such as the hypothalamus and cortex (Figure S11), with the uptake beginning within 1–2 h post-injection and persisting for over 24 h. We then performed a therapeutic study testing whether administration of htNSC-EVs could influence neurological outcomes in PTMS-KO mice. We compared EVs containing or missing PTMS, released by cultured htNSCs from the WT and PTMS-KO model, respectively. Middle-aged PTMS-KO animals were treated with these EVs, while vehicle treatment was included as the basal control. EVs were administered through pre-implanted cannula in the hypothalamic third ventricle, at the dose of 100 ng per injection, 3 times per week, for a duration of 3 months. Neurobehavioral assessments were finally performed to evaluate physical, cognitive, and social function. As shown in Figures 6A and 6B, treatment with htNSC-derived EVs led to improvements in neurological functions, although not to normal levels, whereas EVs lacking PTMS were unable to produce or less effective in producing similar benefits. At the end of the experiment, brain tissues were collected and analyzed for DNA damage using pH2A.X immunostaining. Consistently, htNSC-derived EVs significantly reduced, although did not fully prevent, DNA damage across multiple brain regions, while PTMS-missing EVs were insufficient to provide this protection (Figures 6C–6E). In summary, the neurological deficits observed in the PTMS-KO model are partially reversed by restoring htNSC-derived EVs in a manner that depends on the presence of PTMS cargo.

### Therapeutic potential of PTMS-containing htNSC-EVs in the 5xFAD model

**Figure 7.**
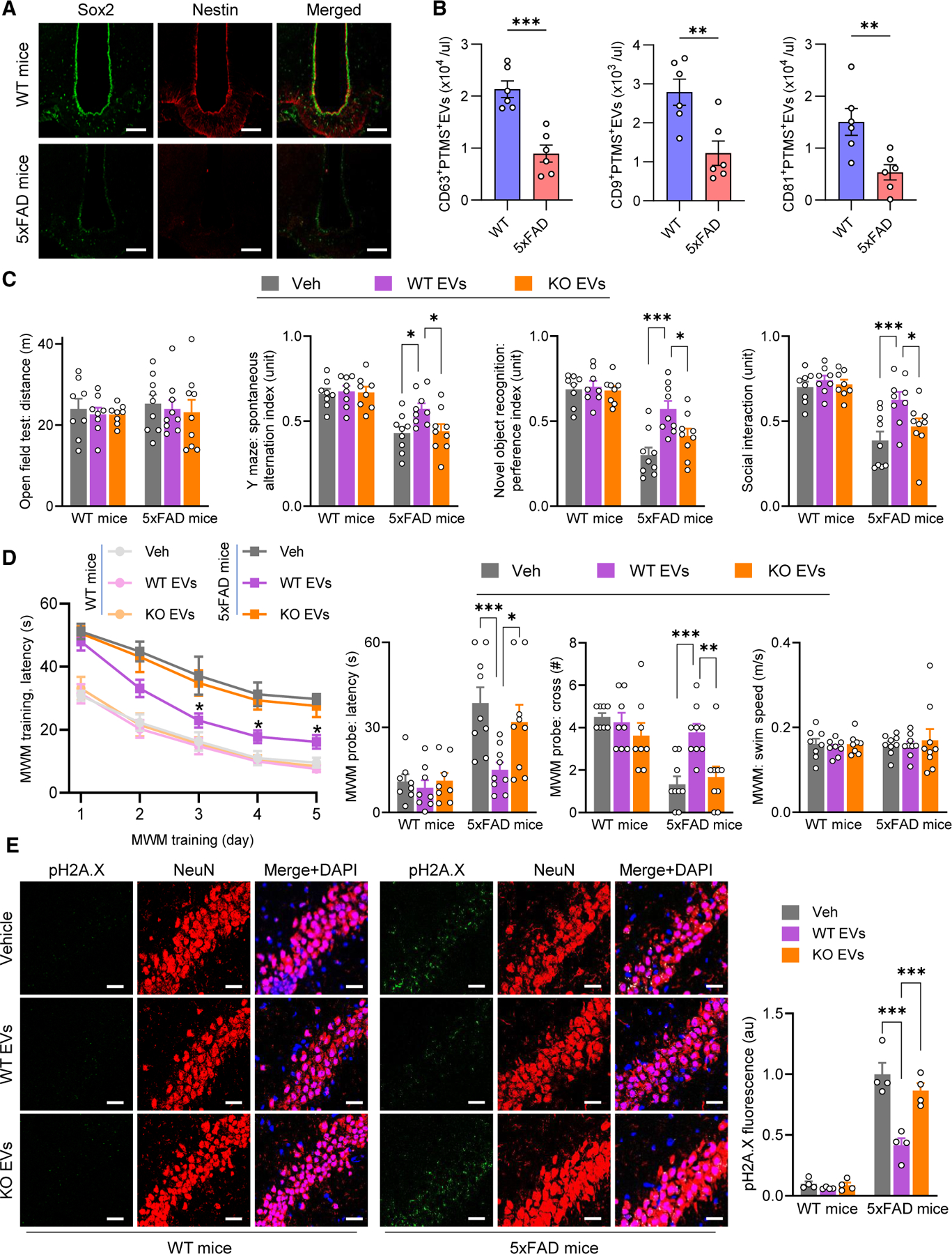
Therapy against neurodegeneration in the 5xFAD model by htNSC EVs (A) Brain sections from 5xFAD mice and littermate WT controls (5 months old, male) were immunostained for neural stem cell markers Sox2 and Nestin in the MBH. Scale bars, 100 μm. (B) The CSF samples from 5xFAD mice and littermate WT controls (5 months old, male) were analyzed for PTMS-positive EVs, including quantification of total EVs and subsets expressing tetraspanins (CD9, CD63, CD81). (C–E) 5xFAD mice and littermate WT controls (5 months old, male) were treated intranasally on a daily basis for 6 weeks with either vehicle or 1 μg purified EVs from two htNSC versions, established from newborn WT mice (labeled as WT EVs) and PTMS-KO mice (labeled as KO EVs), respectively. Vehicle (Veh) treatment was included for basal control. During the final 2 weeks of treatment, mice underwent cognitive testing, including a behavioral battery (C) and Morris water maze (MWM) (D). Subsets of mice were used for immunostaining of DNA damage marker pH2A.X (E), showing representative images of the hippocampal CA3 subregions followed by quantification. Scale bars, 25 μm. Statistics: *p < 0.05, **p < 0.01, ***p < 0.001, two-tailed unpaired Student’s *t* test (B), and two-way ANOVA with Tukey’s post hoc test (C–E); *n* = 6 mice per group (B), *n* = 8–9 mice per group (C and D), and *n* = 4 mice per group (E). Statistical *p* values in the curve graph (D, left) represent comparisons between WT EVs and each of the other two groups within the 5xFAD model at the matched time points. Data are presented as mean ± SEM. See Figures S12 and S13 for additional information.

To extend this study to aging-related neurodegeneration, we investigated the therapeutic potential of htNSC-EVs against AD using the 5xFAD mouse model as a proof-of-concept study. This approach was further supported by evidence showing that by mature-adult age, 5xFAD mice already have a reduced htNSC population compared to WT controls (Figure 7A). We also appreciated that PTMS-positive EVs in the cerebrospinal fluid (CSF) were less abundant in 5xFAD mice compared to WT controls (Figure 7B). For the therapeutic study, the treatment was initiated at 5 months of age, a time point at which 5xFAD mice already exhibit significant cognitive deficits and neurodegenerative pathology, whereas WT controls remain phenotypically normal. To determine the role of PTMS in mediating therapeutic effects, we continued to compare EVs containing or missing PTMS, released by cultured htNSCs obtained from WT and PTMS-KO model, respectively. To enhance translational relevance, we employed intranasal administration, a non-invasive route amenable to repeated dosing and more applicable in clinical settings. This approach is further supported by recent studies demonstrating brain delivery of EVs via the nasal route.^21–24^ To justify this delivery route, we compared therapeutic doses administered intranasally versus ICV administration, showing that intranasal delivery is comparable and even more effective in targeting outer brain regions such as the olfactory bulb and cortex (Figure S12). Thus, we implemented a daily intranasal administration regimen for treating 5xFAD model and WT controls, using htNSC-EVs containing or missing PTMS, and vehicle was used as the basal control. At the end of the 6-week treatment, mice were assessed for neurological functions. Results showed that treatment with htNSC-EVs led to significant improvements in cognitive and social functions in 5xFAD mice, in a manner dependent on the presence of PTMS (Figures 7C and 7D). The neurobehavioral functions were still normal in WT mice under this study, which were barely affected by either EV treatment. At the end of this study, brain tissues were collected and assessed for neuronal DNA damage. Consistent with behavioral findings, htNSC-EVs markedly reduced DNA damage in the brain, while these effects compromised when PTMS was missing in these EVs (Figures 7E and S13). In summary, htNSC EVs bear a therapeutic potential in an AD-like model, while PTMS plays an important role in mediating these therapeutic effects.

## DISCUSSION

In this study, as schematized in Figure S14, we propose PTMS as a key brain-derived factor essential for counteracting neurodegeneration. Using mouse models, we show that PTMS genetic loss of function leads to severe age-related neurodegenerative pathology and reduced lifespan, whereas hypothalamic PTMS gain of function alleviates age-related neurological deficits and neuronal DNA damage, accompanied by lifespan extension. We further demonstrate that specific subpopulations of htNSC-derived EVs carry PTMS in association with sn/snoRNAs and work to deliver the complex of these cargos into neurons to protect against neuronal DNA damage. Therapeutically, using PTMS-containing htNSC-EVs represents a promising strategy for targeting aging-related neurodegeneration and AD-like disorders in mouse models.

Although PTMS can be produced by various hypothalamic and extra-hypothalamic cell types such as neurons, htNSCs are particularly significant from the brain-wide neurocrine perspective, considering that these cells are mainly located along the third ventricle wall, which allows the release of htNSC EVs directly into the CSF for widespread distribution throughout the brain. Despite a relatively small population of htNSCs, the steady-state release of PTMS-containing EVs from these cells appears to constitute an essential level of support to brain and neuronal health. Overall, our findings support a conceptual framework that htNSC-EVs are an important component in the neuroprotective secretome of these cells, while supplementation with these EVs offers a promising strategy to combat neurodegeneration. As the therapeutic relevance of stem-cell-derived EVs gains increasing attention, including recent efforts in targeting AD models,^25,26^ future studies should aim to compare EVs from htNSCs with those from other sources and explore the potential for synergistic applications.

Our study reveals that the anti-neurodegenerative effects of htNSC-derived EVs are linked to their capacity to attenuate neuronal DNA damage, a process that requires the presence of PTMS cargo. This seems particularly important for neurons, which are post-mitotic and thus are constantly influenced by cumulative genomic insults. Although the precise mechanisms by which PTMS confers DNA protection remain to be elucidated, we observed that PTMS is associated with sn/snoRNAs within EVs, a class of small RNAs implicated in genome stability and stress responses.^17–20^ Future studies dissecting the roles of sn/snoRNAs will be valuable, especially as technologies emerge to selectively edit individual small RNA species within EVs. It is also necessary to note that, while PTMS is essential for the neuroprotective effects of htNSC-derived EVs, it is unlikely to be the sole protein effector; other protein and peptide cargos most likely also contribute. Comprehensive profiling of these EVs and comparative functional studies with other EV types represent important directions for future research.

An interesting question arising from this work is whether cross-talk exists between hypothalamic EVs and secretory peptides in the brain to mediate anti-neurodegenerative effects. To address this question represents a promising area for future investigation. Our recent research showed that a combination of hypothalamic reproductive neuropeptides exerts neuroprotective effects in the 5xFAD model.^27^ Additionally, the CSF contains various neuroprotective factors such as Fgf17, which supports oligodendrogenesis.^28^ We hypothesize that neuropeptides and hypothalamic EVs can interact closely and may promote each other, forming a regulatory network ultimately responsible for optimal effects in counteracting neurodegeneration. Given the multifaceted properties and pharmacokinetic advantages of EVs, they represent a significant and at least complementary strategy for developing therapies against neurodegenerative diseases.

Toward clinical translation, we evaluated the intranasal administration of htNSC-derived EVs in the 5xFAD model and demonstrated the feasibility of this non-invasive delivery route for targeting the brain, resulting in neuropathological and cognitive improvements. Nevertheless, key challenges remain, including the generation of clinically compatible, htNSC-derived EVs of human origin. Although animal-derived EVs may be tolerated due to the low immunogenicity of stem-cell-derived products for application, human htNSC-like cells could potentially be generated via induced pluripotent stem cell reprogramming, as demonstrated in previous studies.^29–31^ Ongoing progress in stem cell reprogramming and EV bioengineering is expected to facilitate the clinical translation of this platform for treating neurodegenerative diseases.

### Limitations of the study

Several limitations of this study should be acknowledged. First, although we highlight the therapeutic potential of PTMS-containing hypothalamic EVs derived from htNSCs, PTMS is also expressed and released by multiple brain cell types, including various neuronal populations in both hypothalamic and other brain regions. These additional sources are likely to also contribute to the neuroprotective effects of PTMS; however, this study did not explore how PTMS is released by other types of neural cells or how it contributes to neuroprotection locally versus systemically within the brain. Second, while our findings suggest that PTMS protects against neuronal DNA damage, presumably involving sn/snoRNAs, the underlying mechanisms of PTMS-mediated neuroprotection still remain unclear. Finally, we did not investigate the potential role of peripheral PTMS, such as circulating PTMS in specific secretory forms, which may cross into the brain and exert neuroprotective effects. Addressing these limitations in future studies will be critical to clarify the contributions of PTMS from different cellular sources, their release mechanisms, and whether they rely on EVs or alternative delivery pathways.

## RESOURCE AVAILABILITY

### Lead contact

Requests for further information and resources should be directed to and will be provided by the lead contact, Dongsheng Cai (dongsheng.cai@einsteinmed.edu).

### Materials availability

All materials in this paper will be available from the lead contact upon request.

### Data and code availability

- All data reported in this paper will be available from the lead contact upon request.
- This paper does not involve generating any original code.
- Any additional information in this paper is available from the lead contact upon request.

## STAR★METHODS

### EXPERIMENTAL MODEL AND STUDY PARTICIPANT DETAILS

Cell culture models: Primary culture of htNSCs was performed as we performed previously.^2,9,10,15,33^ In brief, the hypothalamic tissue was dissected from neonatal C57BL/6 mice, cut into small pieces and followed by 10-min digestion using TrypLE Express enzyme (Life Technologies) at 37°C. After centrifugation, cells were suspended in the neurobasal-A and B27 medium (NA/B27) medium containing neurobasal-A (Life Technologies), 2% B27 without vitamin A (Life Technologies), 10 ng/mL EGF (Sigma-Aldrich), 10 ng/mL bFGF (Life Technologies), 0.25% GlutaMAX supplement (Life Technologies), and 1% penicillin-streptomycin, and were cultured under 37°C and 5% CO_2_. One week later, neurospheres were collected by centrifugation and trypsinized into single cells and continued to be sub-cultured in neurospheres, while NA/B27 medium was replaced every 2 days to remove debris. Through serial passages, the htNSC population was selectively enriched and expanded through forming neurospheres and were established after 4 to 5 passages. The identity of htNSCs was confirmed by immunostaining for neural stem cell biomarkers, including Sox2 and Nestin. Additionally, immunostaining for biomarkers of neurons, astrocytes, microglia, and oligodendrocytes confirmed the absence of these cell types in htNSC cultures. Neuronal models used in this study included the generic neuronal cell line Neuro-2A (ATCC) and two hypothalamic neuronal cell lines, mHypoA-2/21 (Cedarlane) and GT1–7 ^4^. This study also included using HEK293T cell line (ATCC) as a tool of producing recombinant lentiviruses. All neuronal models and HEK293T cells were cultured in DMEM supplemented with 10% fetal bovine serum and 1% penicillin-streptomycin, maintained at 37°C in a 5% CO_2_ atmosphere. All cell lines were confirmed free of microbial and mycoplasma contamination, and morphology and growth characteristics of cells were consistent with published descriptions and authenticated.

Animal models: Founder PTMS knockout (PTMS-KO) mice were generated at Einstein’s transgenic core using CRISPR technology^34^ and were briefly introduced in our previous work for PTMS antibody validation.^5^ In detail, two guide RNAs (gRNAs) were designed: Ptms gRNA1 targeting Intron 1 of the Ptms gene (sequence: ggtgggagcctcagcgactctgg) and Ptms gRNA2 targeting a downstream region following exon 5 (sequence: tgatatagtgtgcactgctgagg). These gRNAs were produced by *in vitro* transcription. For founder generation, two single-stranded homologous donor DNAs, Cas9 protein, and the Ptms gRNAs were injected into fertilized C57BL/6 mouse eggs. The injected eggs were then transferred to pseudo-pregnant CD1 female mice to produce pups. Animals were screened for PTMS knockout founders by PCR and sequencing to confirm excision of Ptms exons 2 to 5. Founder PTMS-KO was then bred with standard C57BL/6 mice to produce the F1 generation, and the line has been maintained on the C57BL/6 background. Heterozygous progenies were interbred to generate homozygous KO mice and genetically matched littermate WT controls for experiments. 5xFAD mouse strain and standard C57BL/6 mice were obtained from Jackson Laboratory and expanded from breeding colonies at Albert Einstein College of Medicine. For PTMS KO and wildtype control mice, both sexes were studied from young ages (3 months old) until old ages (24 months old) while lifespan assays spanned the entire lifespan. For 5xFAD strain and wildtype controls, male models were studied starting from the age of 5 months. Standard male C57BL/6 mice at ∼15 months of age were used to generate hypothalamic PTMS gain-of-function model and control. All animal protocols were approved by the Institutional Animal Care and Use Committee of the Albert Einstein College of Medicine (#00001111, 00001385, 00001397, 00001398, 00001399, and their previous versions).

### METHOD DETAILS

#### Brain procedures

Delivery of lentiviruses into the MBH region and the hypothalamic 3V wall was performed, using our established protocols. MBH delivery was directed at the coordinates of −1.7 anterior-posterior and −5.8 dorsal-ventral from the bregma and at 0.25 lateral from the middle-line at each hemisphere. The hypothalamic 3V delivery was directed at the coordinates of −1.7 anterior-posterior and −5.0 dorsal-ventral from the bregma and at the middle line of the hypothalamus. Lentiviruses were suspended in artificial CSF (aCSF) and injected under an ultra-precise stereotactic apparatus (David Kopf Instruments) via a 26-gauge guide cannula and a 33-gauge internal injector (Plastics One) connected to a 5-μL Hamilton syringe and infusion pump. The volume was 0.2–0.4 μL per site for MBH tissue injection and 0.5 to 1.0 μL for hypothalamic 3V injection, and aCSF was used as the vehicle. For the procedure of CSF sampling, mice were placed on to ultra-precise stereotactic apparatus (David Kopf Instruments) under 2–3% isoflurane anesthesia, and CSF was collected under microscope through the exposed cisterna magna using a bent 33G needle with a tube connected to 1-mL syringe. Animal treatment: animals were treated with ultracentrifugation-purified EVs through i.c.v. or nasal administration at a frequency and for a duration specified in each experiment. For i.c.v. treatment, a mouse received an injection of 100 ng purified EVs suspended in 0.5 μL aCSF or the vehicle as a control through an injection cannula pre-implanted into the third-ventricle space of the hypothalamus. For nasal administration, 1 μg purified EVs suspended in 2 μL saline or the vehicle as a control was delivered into the nostril of a mouse. All animal procedures were approved by the Institutional Animal Care and Use Committee of the Albert Einstein College of Medicine.

#### EV assays

EVs were isolated and purified through the method of filtration and ultracentrifugation, as described in our previous work.^9,10^ EV-free media (through ultracentrifugation) were used in cell culture for releasing EVs. In brief, EVs were pre-cleared by centrifugation at 2,000*g* 10 min, followed by filtration with 0.8-μm-pore-size filter (Corning) and purified through sucrose-cushion ultracentrifugation at 110,000*g* 90 min. The quantity of EVs was assessed using Pierce BCA protein assay (Thermo Fisher Scientific) and particle counting (NanoSight LM10 system, Malvern Instruments Ltd). For nano-flow cytometry, purified EVs were incubated with fluorescence-conjugated antibodies and processed at the Flow Cytometry Core at Albert Einstein College of Medicine. The assay included PE-conjugated CD63 antibody (BD biosciences, cat# BDB564222), PE/Dazzle 594-conjugated CD9 (Biolegend, cat# 124822), PE/Cyanin7-conjugated CD81 antibody (Biolegend, cat# 104914), and PTMS antibody (Pierce Biotechnology)^5^ which was conjugated with APC (Lightning-Link, abcam, cat# ab201807). EV samples underwent multiple cycles of switching between room temperature and ice incubation to enhance antibody penetration, followed by 1-h antibody incubation on ice in darkness. Isotype controls staining with APC-conjugated rabbit IgG isotype control (Cell signaling technology, cat# 12445s), PE-conjugated rat IgG2α, κ isotype control (BD biosciences, cat# BD554689), PE/Dazzle 594 rat IgG2α, κ isotype control (Biolegend, cat# 400557), and PE/Cyanin7 conjugated armenian hamster IgG isotype control (Biolegend, cat# 400921) were used to assess non-specific binding. Besides isotype and unstained technical controls, buffer-only, buffer-plus-antibody, and 0.1% Triton X-100 detergent control were included for purity assessment under consistent conditions. The threshold for side scatter (SSC) and forward scatter (FSC) were set to 1000, and the gain FSC, SSC, SSC-B, B2 (FITC), R1 (APC), YG1 (PE), YG3 (PE/Dazzle 594) and YG9 (PE/Cyanin7) detectors were set to 532, 1500, 138, 288, 137, 288, 217 and 328, respectively, with the aid of Apogee sizing beads (Apogee Flow Systems, cat #1527). Apogee beads contain a mixture of various reference size beads which were detected with gating strategy, and each bead population was separated from the noise population along with the SSC axis and B2 axis. Buffer control was used to calculate background noise. The same volume of samples was recorded for all samples with low flow rate (up to 15 μl/min) to minimize the swarming effect. Data was collected, analyzed and compensated using SpectroFlo software. The compensation was adjusted with unstained and single-color controls by the spectral unmixing, which considers the entire spectrum of fluorescence to distinguish the overlapping signals from different fluorescent channels. FlowJo software was used to generate plot images. EV labeling with PKH26 lipophilic dye (Sigma-Aldrich) was performed according to the previously reported method with slight modifications.^35^ Lipophilic dye-labelled EVs were purified through sucrose-cushion ultracentrifugation. For immunostaining, purified EVs were applied to a coverslip, blocked with the serum of appropriate species, treated with primary antibodies, including rabbit anti-PTMS (Pierce Biotechnology),^5^ rabbit anti-HA (Cell Signaling, 3724), rat anti-Sox2 (R&D Systems, MAB2018), mouse anti-CD9 (Santa Cruz, sc-13118), mouse anti-CD63 (Santa Cruz, sc-5275), mouse anti-CD81 (Santa Cruz, sc-23962), subsequently incubated with Alexa Fluor 488 or 555-conjugated secondary antibodies. Technical controls included naive IgGs of the appropriate species. Images were captured using Leica SP8 confocal microscope.

#### Plasmids and recombinant lentiviruses

Plasmid plenti-CMV-*ptms*-HA was obtained by inserting coding sequence of *ptms*-HA into the plenti6 backbone (Invitrogen) with *BamHI* and *XhoI* sites. HA was tagged at the C-terminal of PTMS to protect its N-terminal for secretion of this protein. The size-matched control sequence was based on scrambled version of *ptms* which was synthesized and verified by Genewiz and was inserted into the plenti6 backbone to generate plenti-CMV-control-HA. The HA-tagged scrambled control sequence of *ptms*: 5-GGATCCGCCACCATGCGCGAAGAAAAAGAAGAT GAAAAAGATGAAGATGCGATGCTGAAACCGGAAGATAACGGCGAAGAAAAAGAAGCGGATGAAGTGGAAGGCGATACCGCGAAAGAAGATGAAACCCTGGCGGAAGCGGAACGCAGCGAAAAAGAAAAAGAAGAAGGCGCGGCGGAAGAAGCGGATGAACGCGAAAAAGAAAAAGAAGAAAGCGAACGCACCGTGGATGAAGAAAACGAAAAAGAACAGAAAGGCAGCCCGGAAAGCAAAGCGAAAGAAGCGGGCGATGCGCGCGTGGTGGGCGTGGAAGAAGGAAGCGGATACCCATACGATGTTCCAGATTACGCTTGACTCGAG-3. Synapsin promoter-directed lentiviral vector was obtained by inserting *ptms* or its scrambled control sequence into the Synapsin lentiviral plasmid with *BamHI* and *PciI* sites. Lentiviruses were produced by transfecting HEK293T cells with corresponding viral and packaging plasmids, purified by ultracentrifugation and titrated using p24 ELISA kit.

#### Western blot

Protein lysate was prepared by sonication in ice-cold lysis buffer (20 mM Tris-HCI, pH 7.4, 10 mM NaCl, 1 mM EDTA, 0.01% SDS, 1% Triton X-100 and 1x protease inhibitor cocktail). Protein samples following heat denaturation were separated by SDS-PAGE and were transferred onto PVDF membranes, which were fixed, blocked and incubated with primary antibodies overnight at 4°C. The assays included rabbit anti-PTMS antibody (1:1000, Pierce Biotechnology),^5^ rabbit anti-HA (1:1000, Cell Signaling, 3724), mouse anti-TSG101 (1:1000, Santa Cruz, sc-7964), mouse anti-CD63 (1:1000, Santa Cruz, sc-5275), mouse anti-CD81 (1:1000, Santa Cruz, sc-23962), mouse anti-Dicer (1:1000, Santa Cruz, sc-136979). and mouse anti-GAPDH (1:1000, Cell Signaling, 97166). After washing 3 times, membranes were incubated with HRP-conjugated anti-rabbit or anti-mouse secondary antibody (1:3000, Cell Signaling) at room temperature for 40 to 60 min, then washed 3 times and finally subjected to ECL reaction for imaging.

#### RNA immunoprecipitation (RIP) and RNA assays

RIP assays were conducted using the EZ-Magna RIP Kit (Merck Millipore 17–700, USA). In brief, pre-equilibrated magnetic protein A/G beads (#88802, Thermo Scientific) and mouse IgG (#CS200621, Sigma) or anti-HA (#2367, Signaling Technology) were mixed and incubated at 4°C on a horizontal shaker overnight. The EVs were obtained from the cell culture media, purified and dissolved in lysis buffer, and incubated with a mixture of magnetic protein A/G beads and mouse IgG or anti-HA at 4°C on a horizontal shaker overnight. Finally, the mixture was spun down and sequentially re-suspended with Proteinase K, TRIzol, chloroform, isopropanol, and 75% ethanol. The final mixture was dissolved in DEPC water, target small RNAs in precipitates were quantified by qRT-PCR after polyadenylation and cDNA synthesis using the Lucigen poly(A) polymerase tailing kit (PAP5104H) and the SuperScript III First-Strand Synthesis System (18080–051; Invitrogen) with a universal RT primer (5′-CAGTGCAGG GTCCGAGGTCAGAGCCACCTGGGCAATTTTTTTTTTTVN-3′). For cellular small RNA levels, small RNAs were isolated from cells using mirVana miRNA isolation kit on the basis of the manufacturer’s instructions (Invitrogen). Real-time qPCR using the SYBR Green PCR Master Mix (Applied Bio-systems) was performed for small RNAs. Relative levels of small RNAs in the cells were normalized according to levels of U6 (forward: 5ʹ-CTCGCTTCGGCAGCACA-3ʹ; reverse: 5ʹ-AACGCTTCACGAATTTGCGT-3ʹ). PCR primer sequences of individual sn/snoRNA species are listed as follows:

Gm22448: 5ʹ-TTAGCATGGCCCCTGAACAA-3ʹ, Gm24407: 5ʹ-TTTATCCGAGGCGCGATTAT-3ʹ, Gm23814: 5ʹ-AAGGATGACACGCAAATTCG-3ʹ, Gm25313: 5ʹ-ATTTCCGTGGAGAGGAACAA-3ʹ, Gm24265: 5ʹ-AGCCAATGAGGTTTATCCGA-3ʹ, Rnu5g: 5ʹ-CAGAGAAGATTAGCATGGCC-3ʹ, Gm25793: 5ʹ-AGAGAAGATTAGCATGGCCC-3ʹ, Gm27607 5ʹ-GGAGGGAGAACAAGTCTAGC-3ʹ, Gm22247: 5ʹ-CTTTGGGACATTGGAATTGG-3ʹ, Gm25461: 5ʹ-CCCAATGTCATGAAGAAAGGT-3, Gm22776: 5′-GATTGCCAGTCAAACATTCC-3′, Gm23787: 5ʹ-CCAAGGTATGAGAGAGATGACG-3ʹ, Gm25133: 5ʹ-TTCCTTTGTCAGTGGGGTCA-3ʹ, Scarna9: 5ʹ-GAATTTTGTCACTGTGAAGGC-3ʹ, Gm22962: 5ʹ-TATGGGGTTTCTGACCAAGG-3ʹ, Gm25087: 5ʹ-GATGACAACCCAATGTCATGA-3ʹ, Gm23723: 5ʹ-GGTAGCAGTTGTAGCATTCC-3ʹ, Snord90: 5ʹ-ATAGGGCAGATTCTGAGGTG-3ʹ, AF357428: 5ʹ-GATCTGATGGTGTCTGAGTG-3ʹ, Gm25635: 5ʹ-GGACATTGAAATTGGCTGAG-3ʹ, Gm24518: 5ʹ-CCCAGTCAAACATTCCTTGG-3ʹ, universal reverse primer: 5′-CAG TGC AGG GTC CGA GGT-3′

#### Neurobehavioral assays

Neurobehavior assays were performed in a specifically designated behavioral testing room with an Anymaze video-tracking system (Stoelting) equipped with a digital camera and computer for recording animal behaviors. (1) The grip test: A mouse was lifted by the tail and placed on a homemade grid (1-cm mesh size), which was then inverted over a pad, and the mouse was allowed to hang for 5 min. Three repeats were performed for each mouse with 10-min rest between any two trials. (2) Open field test: A mouse was placed into a corner of a white plastic chamber (40 cm length, 40 width, and 40 cm height). The mouse was allowed to freely explore the chamber, and locomotion was recorded over a 10-min duration. (3) Novel object recognition test: A mouse was allowed to freely explore an open-field box for 10 min. During familiarization session, the mouse was allowed to freely explore two similar objects, and then during test session, one was replaced by a novel object for the mouse to explore 10 min. Interval time between two sessions was 6 h. The time that the mouse spent exploring each object was recorded. A preference index was calculated using the ratio of time spent exploring novel object over the total time of exploring both objects. (4) Y maze test: The test was performed in a Y-shaped maze with three arms with 120-degree angle from each other. A mouse was placed into one arm facing the center junction, and allowed to freely explore for 10 min. An arm entry occurred when all 4 paws were confirmed to cross the arm-center zone boundary with the snout toward the end of an arm. Y-maze index was defined as the number of spontaneous alternations divided by the total number of arm entries. (5) Social function test: The test was performed in a plastic three-chamber neutral box cage (60 cm length, 40 cm width, and 22 cm height). Each mouse was allowed to freely explore for 5 min to adapt the testing conditions. During social affiliation session, a new mouse (stranger 1) was placed in a wire containment cup that was located in one side chamber. Subject mouse was allowed to freely access and explore each of the three chambers for 10 min. During social recognition session, a second new mouse (stranger 2) was placed in a wire containment cup and put in the opposite side chamber. The subject mouse freely explored each of the three chambers for 10 min. The time spent in each chamber and time spent sniffing were recorded. Social exploring index was calculated as the ratio of the time spent in the chamber containing stranger 2 to the total time. Social interaction index was calculated as the ratio of sniffing time with stranger 2 to the sniffing time with both strangers. (6) Morris water maze (MWM) test: The test was performed using a water tank that was filled with 22–23°C water, crayola non-toxic paint was added to make opaque and white background. The maze with 90 cm in diameter was virtually divided into four quadrants. A circular, background color-matched platform with a diameter of 10 cm was placed 25 cm from the wall of the tank, 0.5 to 1 cm below the water surface, so it was invisible to the animals under test. Animals were trained for 4 or 5 days, 2 training sessions each day, and the starting positions of each training day were semi-randomly chosen. On each training day, mice were placed in the desired starting position in the maze and were trained to find the platform within 60 s, guided by a glass stirring rod when necessary. The latency to reach the platform of each trail was recorded. Spatial memory was assessed on the next day following the completion of the training session, by removing the platform, and the animal was allowed to swim for 60 s. The latency and number of times to cross the location of the former platform, and the swimming speed were measured. All animal procedures were approved by the Institutional Animal Care and Use Committee of the Albert Einstein College of Medicine.

#### Histology and biochemistry

Mice under anesthesia were transcardially perfused with PBS and 4% paraformaldehyde, and then brains were obtained, post-fixed and equilibrated with 20–30% sucrose, and cryosectioned at 10–30 μm thickness, with consistent thickness used within each experiment. Brain sections were blocked with the serum of appropriate species, treated with primary antibodies at 4°C overnight, followed by reaction with fluorescent secondary antibodies, with 3 washes performed after each step. Primary antibodies included rabbit anti-PTMS (Pierce Biotechnology),^5^ rabbit anti-HA (Cell Signaling, 3724), rabbit anti-phospho-Histone H2A.X (Cell Signaling, 9718), rabbit anti-Sox2 (R&D Systems, MAB2018), mouse anti-NeuN (Millipore, MAB377), and mouse anti-HuC/D (Invitrogen, A21271). Secondary antibodies included Alexa Fluor 488 and 555-conjugated IgG. Technical controls included naive IgGs of appropriate species. For PKH26 fluorescence, brain tissues were obtained from animals which received delivery of PKH26-labelled EVs using the same procedure described above and brain sections were used for co-immunostaining. For Fluoro-Jade C (FJC) staining, fluorescent FJC (Sigma) was applied to frozen sections for 10 min at room temperature before washing and immunostaining. DAPI in the mounting medium was used to reveal the nuclei of all cells. Images were captured using confocal microscope. Nissl staining (Sigma) was conducted on frozen brain sections according to the manufacturer’s instruction, and Nissl staining was scanned with PerkinElmer P250 High-Capacity side scanner. For biochemistry, PKH26 fluorescence in tissues of injected mice were measured by generating tissue lysates and examined under a microplate reader at excitation 485 nm and emission 525 nm (Molecular Devices), and the same tissue types from vehicle injection were used to provide noise which was subtracted for calculating fluorescence signal.

### QUANTIFICATION AND STATISTICAL ANALYSIS

All measured data were presented as mean ± SEM. Sample sizes with sufficient power were designed according to our published studies, relevant literature and preliminary studies. For animal treatment studies, animals were randomized and randomly assigned into treatment versus control. All experiments were repeated independently or through complementary approaches. For neurobehavioral assays, an experimental performer was blind to group information before data being recorded, and researchers were arranged to provide independent evaluations on key experiments of testing a hypothesis. Lifespan survey was based on natural death of animals, but when a mouse was under terminal stage suffering from severe complications and was expected to die within a few days or less, euthanasia was used per animal protocols following consultation with attending veterinarians. All numeric values were subjected to parametric analysis. Only parametric data were analyzed using two-tailed unpaired Student’s t-test for 2-group comparisons and using ANOVA for >2-group comparisons followed by post-hoc Tukey’s test only after overall comparisons across all groups were statistically significant. For lifespan analysis, Kaplan–Meier survival curve and log rank (Mantel-Cox) test were used. Software for performing statistics included GraphPad Prism and Excel, and *p* value of less than 0.05 was considered significant (*p < 0.05, **p < 0.01, ***p < 0.001, ****p < 0.0001).

## Supplementary Material

1

Supplemental information can be found online at https://doi.org/10.1016/j.celrep.2025.116561.

## Figures and Tables

**Table:** KEY RESOURCES TABLE

REAGENT or RESOURCE	SOURCE	IDENTIFIER
Antibodies		

PE-conjugated CD63	BD biosciences	cat# 564222; RRID: AB_2738678
PE/Dazzle 594-conjugated CD9	Biolegend	cat# 124822; RRID: AB_2800602
PE/Cyanin7-conjugated CD81	Biolegend	cat# 104914; RRID: AB_2810340
APC-conjugated rabbit IgG	Cell signaling	cat# 12445s; RRID: N/A
PE-conjugated rat IgG2α, κ	BD biosciences	cat# 554689; RRID: AB_479724
PE/Dazzle 594 rat IgG2α, κ	Biolegend	cat# 400557; RRID: AB_2923260
PE/Cyanin7 conjugated armenian hamster IgG	Biolegend	cat# 400921; RRID: AB_2905473
rabbit anti-PTMS	Pierce Biotechnology	Previous study^5^
rabbit anti-HA	Cell Signaling	cat# 3724; RRID: AB_1549585
mouse anti-HA	Cell Signaling	cat# 2367; RRID: AB_10691311
rat anti-Sox2	R&D Systems	cat# MAB2018; RRID: AB_358009
mouse anti-CD9	Santa Cruz	cat# sc-13118; RRID: AB_627213
mouse anti-CD63	Santa Cruz	cat# sc-5275; RRID: AB_627877
mouse anti-CD81	Santa Cruz	cat# sc-23962; RRID: AB_627192
mouse anti-TSG101	Santa Cruz	cat# sc-7964; RRID: AB_671392
mouse anti-Dicer	Santa Cruz	cat# sc-136979; RRID: AB_2230615
mouse anti-GAPDH	Cell Signaling	cat# 97166; RRID: AB_2756824
rabbit anti-phospho-Histone H2A.X	Cell Signaling	cat# 9718; RRID: AB_2118009
mouse anti-NeuN	Millipore	cat# MAB377; RRID: AB_2298772
mouse anti-HuC/D	Invitrogen	cat# A21271; RRID: AB_221448
Alexa Fluor 488 goat anti mouse	Invitrogen	cat# A28175; RRID: AB_2536161
Alexa Fluor 488 goat anti rabbit	Invitrogen	cat# A11008; RRID: AB_143165
Alexa Fluor 488 goat anti rat	Invitrogen	cat# A11006; RRID: AB_2534074
Alexa Fluor 555 goat anti mouse	Invitrogen	cat# A21422; RRID: AB_141822
Alexa Fluor 555 goat anti rabbit	Invitrogen	cat# A21428; RRID: AB_141784
Alexa Fluor 555 goat anti rat	Invitrogen	cat# A21434; RRID: AB_141733
HRP-conjugated anti-rabbit	Cell Signaling	cat# 7074; RRID: AB_2099233
HRP-conjugated anti-mouse	Cell Signaling	cat# 7076; RRID: AB_330924

Bacterial and virus strains		

pLenti6-CMV-ptms-HA	Our inventory	Previous study^5^
pLenti6-CMV-control-HA	Our inventory	Previous study^5^
pLenti6-Syn-ptms	Our inventory	Previous study^5^
pLenti6-Syn-control	Our inventory	Previous study^5^

Chemicals, peptides, and recombinant proteins		

mouse IgG	Sigma Aldrich	cat# CS200621
PKH26 lipophilic dye	Sigma Aldrich	cat# MINI26–1KT
ECL	Thermo Scientific	cat# 34095
SYBR Green PCR Master Mix	Applied Biosystems	cat# 4309155
Fluoro-Jade C staining kit	Sigma Aldrich	cat# AG325

Critical commercial assays		

Pierce BCA protein assay	Thermo Scientific	cat# 23225
Lightning-Link APC conjugation kit	Abcam	cat# ab201807
EZ-Magna RNA immunoprecipitation Kit	Merck Millipore	cat# 17–700
magnetic protein A/G beads	Thermo Scientific	cat# 88802
Lucigen poly(A) polymerase tailing kit	Invitrogen	cat# PAP5104H
SuperScript III First-Strand Synthesis System	Invitrogen	cat# 18080–051
mirVana miRNA isolation kit	Invitrogen	cat# AM1561

Experimental models: Cell lines

htNSCs	Primary culture	Previous study^2,5,12^
Neuro-2A	ATCC	cat# CCL-131; RRID: CVCL_0470
mHypoA-2/21	Cedarlane	cat# CLU181; RRID: N/A
GT1–7	Obtained from P. Mellon	Previous study^32^
HEK293T	ATCC	cat# CRL-3216; RRID: CVCL_0063

Experimental models: Organisms/strains		

C57BL/6J	Jackson lab	cat# 000664; RRID: IMSR_JAX:000664
PTMS-KO mouse	Einstein’s transgenic core	Previous study^5^
5xFAD	Jackson lab	cat# 034848; RRID: MMRRC_034848-JAX

Oligonucleotides		

Universal RT primer	Our inventory	CAGTGCAGG GTCCGAGGTCAGAGCCACCTGGGCAATTTTTTTTTTTVN
U6-F	Our inventory	CTCGCTTCGGCAGCACA
U6-R	Our inventory	AACGCTTCACGAATTTGCGT
Gm22448-F	Our inventory	TTAGCATGGCCCCTGAACAA
Gm24407-F	Our inventory	TTTATCCGAGGCGCGATTAT
Gm23814-F	Our inventory	AAGGATGACACGCAAATTCG
Gm25313-F	Our inventory	ATTTCCGTGGAGAGGAACAA
Gm24265-F	Our inventory	AGCCAATGAGGTTTATCCGA
Rnu5g-F	Our inventory	CAGAGAAGATTAGCATGGCC
Gm25793-F	Our inventory	AGAGAAGATTAGCATGGCCC
Gm27607-F	Our inventory	GGAGGGAGAACAAGTCTAGC
Gm22247-F	Our inventory	CTTTGGGACATTGGAATTGG
Gm25461-F	Our inventory	CCCAATGTCATGAAGAAAGGT
Gm22776-F	Our inventory	GATTGCCAGTCAAACATTCC
Gm23787-F	Our inventory	CCAAGGTATGAGAGAGATGACG
Gm25133-F	Our inventory	TTCCTTTGTCAGTGGGGTCA
Scarna9-F	Our inventory	GAATTTTGTCACTGTGAAGGC
Gm22962-F	Our inventory	TATGGGGTTTCTGACCAAGG
Gm25087-F	Our inventory	GATGACAACCCAATGTCATGA
Gm23723-F	Our inventory	GGTAGCAGTTGTAGCATTCC
Snord90-F	Our inventory	ATAGGGCAGATTCTGAGGTG
AF357428-F	Our inventory	GATCTGATGGTGTCTGAGTG
Gm25635-F	Our inventory	GGACATTGAAATTGGCTGAG
Gm24518-F	Our inventory	CCCAGTCAAACATTCCTTGG
universal reverse primer	Our inventory	CAGTGCAGG GTCCGAGGT

Software and algorithms

SpectroFlo	Cytekbio	https://cytekbio.com/pages/spectro-flo
FlowJo	BD Biosciences	https://www.flowjo.com/
Anymaze	ANY-maze	https://www.any-maze.com/
ImageJ	NIH	https://imagej.net/ij/
Graphpad prism	Prism	https://www.graphpad.com/
BioRender	BioRender	https://www.biorender.com/

## Highlights

- PTMS deficiency drives age-related neurodegeneration and reduces lifespan
- Hypothalamic PTMS gain of function mitigates neurodegeneration and prolongs lifespan
- Certain hypothalamic EVs are neuroprotective in a PTMS-dependent manner
- Certain hypothalamic EVs can treat Alzheimer’s disease in a PTMS-dependent manner
