# Supplementary material for: Control of aging-associated neurodegeneration via hypothalamic extracellular vesicles containing parathymosin
Source: Cell Rep. Author manuscript; Available in PMC 2026 Feb 21. (PMC12924715; doi:10.1016/j.celrep.2025.116561)
Supplement: 1 [file NIHMS2125832-supplement-1.pdf]

Cell Reports, Volume *44*

Supplemental information

Control of aging-associated neurodegeneration via hypothalamic extracellular vesicles containing parathymosin

Hyun-Gug Jung, Bin Yu, Yuna Choi, Gyeongyun Go, Qichao Zhang, Yizhe Tang, Min Woo Kim, and Dongsheng Cai

Supplementary Document

Supplemental Figure legends

Suppl. Figure 1. Genotypic validation of the PTMS-KO mouse model.

(A) Schematic representation of the Ptms gene targeting strategy. Approximately 1.5 kb of genomic DNA spanning exons 2 to 5 was excised to generate the KO allele. **(B)** Genotyping by PCR confirmed successful deletion in PTMS-KO mice compared to WT littermates. Related to STAR methods.

Suppl. Figure 2. Neurobehaviors of young PTMS-KO and WT mice.

PTMS-KO mice and littermate WT controls at a young age (3 months old) based on male (**A, B**) and female (**C, D**) were evaluated using the grip strength test (**A, C**) and Morris Water Maze (MWM) test (**B, D**). Statistics: two-tailed unpaired Student's t-test (grip strength, OFT, and MWM probe test); one-way ANOVA with Tukey's post hoc test (MWM training); $n = 15$ mice per group; data are presented as mean ± SEM. Related to Figure 1.

Suppl. Fig 3. Morris Water Maze training in aged PTMS-KO and WT mice.

Male and female PTMS-KO mice and their littermate WT controls underwent Morris Water Maze (MWM) training sessions prior to the probe test shown in Figure 1. This suppl. figure presents performance data during the training phase. Statistics: $*p < 0.05$, two-way ANOVA with post hoc test; $n = 15$ mice per group; data are presented as mean ± SEM. Related to Figure 1.

Suppl. Fig 4. Brain histology in young-adult PTMS-KO and WT mice.

Nissl staining of brain sections from male PTMS-KO mice and littermate WT controls at a young adult age (4 months old). Representative images are shown. Scale bar: 200 μm. Related to Figure 2.

Suppl. Figure 5. Quantification of brain staining in PTMS-KO and WT control.

Quantitative analysis of Fluoro-Jade C (FJC) staining and pH2A.X immunostaining shown in Figure 2D–E. Statistics: $*p < 0.05$, $**p < 0.01$, $***p < 0.001$, two-tailed unpaired Student's t-test; $n = 4$ mice per group; data are presented as mean ± SEM. Related to Figure 2.

Suppl. Figure 6. Site-specific lentiviral delivery targeting the MBH.

Brain images illustrating the anatomical specificity of lentiviral delivery in the hypothalamic PTMS gain-of-function model described in Figure 3A. (A) Low-magnification view of the hypothalamic region containing HA immunostaining directed to the mediobasal hypothalamic subregion surrounding the 3V. (B) Representative extra-hypothalamic brain regions for HA immunostaining and NeuN co-immunostaining, confirming absence of off-target expression relative to the hypothalamic site. Scale bar, 100 μm. Related to Figure 3.

Suppl. Figure 7. Additional assessment of htNSC EVs containing HA-tagged PTMS.

EVs were harvested from htNSCs stably expressing HA-tagged PTMS, purified, and processed for immunostaining of canonical EV tetraspanin markers CD9, CD63, and CD81. Co-localization of HA signal with these markers confirms incorporation of HA-tagged PTMS into the EV subpopulations. Scale bar, 1 μm. Related to Figure 4.

Suppl. Figure 8. Additional analysis of PTMS-sn/snoRNA association in htNSC EVs.

An in-vitro model of Neuro-2A (N2A) neuronal cells and htNSCs were transduced with lentiviruses expressing HA-tagged PTMS. Equal amounts of EVs from each model were subjected to HA immunoprecipitation followed by qPCR profiling for candidate sn/snoRNAs. Statistics: $^{**}p < 0.01$, $^{***}p < 0.001$; two-tailed unpaired Student's t-test; $n = 4$ independent biological replicates per group; data are presented as mean ± SEM. Related to Figure 5.

Suppl. Figure 9. Assessment on htNSC EVs with PTMS or small RNA loss of function.

(A, B) PTMS-negative and PTMS-positive htNSC EVs were isolated from htNSC models originating from PTMS knockout (KO) mice and littermate wild-type (WT) controls, respectively. Equal amounts of purified EVs were analyzed for size distribution using nanoparticle tracking analysis (**A**) and for the expression of EV surface and luminal markers by western blotting (**B**). **(C, D)** Small RNA-deficient htNSC EVs were generated from a hypothalamic neural stem cell (htNSC) line subjected to Dicer knockdown (KD) via lentiviral delivery of shRNAs targeting Dicer. Matched control htNSC line was generated via treatment with lentiviral scramble shRNA (Con), producing control htNSC EVs. The efficiency of Dicer knockdown was validated by Western blotting (**C**) and the consequent reduction of small RNAs was confirmed by quantitative PCR (**D**). Statistics: $^{****}p < 0.0001$; two-tailed unpaired Student's t-test; $n = 3$ independent biological replicates per group; data are presented as mean ± SEM. Related to Figure 5.

Suppl. Figure 10. PKH26-based tracking assay for brain uptake of htNSC EVs.

A therapeutic dose of htNSCs-derived EVs (100 ng) was labeled with fluorescent dye PKH26 and stereotactically injected into 3V of mice through an injection cannula. At 6 hours post-injection, animals were transcardially perfused with saline, followed by fixation. Brain sections were collected, post-fixed, sectioned, washed, and imaged for PKH26 fluorescence. Vehicle-injected animals served as negative controls to verify signal specificity. Representative images are subregions of the mediobasal hypothalamus (MBH), dentate gyrus (DG) of the hippocampus, and parietal cortex. DAPI staining was used to visualize cell nuclei. Scale bar, 25 μm. Related to Figure 6.

Suppl. Figure 11. Time-course analysis of brain uptake of htNSC EVs.

A therapeutic dose of htNSCs-derived EVs (100 ng) was labeled with fluorescent dye PKH26 and stereotactically injected into the 3V of mice using an injection cannula. At 1, 6, and 24 hours post-injection, animals were transcardially perfused with saline, followed by fixation. Brains were post-fixed, sectioned, washed, and imaged for PKH26 fluorescence. Representative images of mediobasal hypothalamus subregion (**A**) parietal cortex subregion (**B**) are shown. HuC/D immunostaining was used to identify neuronal populations. Scale bar, 25 μm. Related to Figure 6.

Suppl. Figure 12. Brain uptake of htNSC EVs via intranasal versus ICV administration.

Mice received either intranasal (1 μg) or intracerebroventricular (ICV; 100 ng) administration of PKH26-labeled htNSCs-derived EVs. ICV delivery was performed through a pre-implanted cannula targeting the 3V. At 2 hours post-administration, mice were transcardially perfused with

saline, and various brain regions were collected for quantification of PKH26 fluorescence using spectrometry. Corresponding brain regions from vehicle-injected mice were used to define background fluorescence. Fluorescence values were normalized to tissue mass. Statistics: **$p < 0.01$; two-tailed unpaired Student's *t*-test; $n = 3$ mice per group; data are presented as mean ± SEM. Related to Figure 7.

Suppl. Figure 13. Additional immunostaining of 5xFAD model following EV therapy.

After the EV therapy protocol, 5xFAD and control WT mice were used for immunostaining of pH2A.X, as described in Fig. 7E. This supplementary panel presents a representative cortex region (parietal cortex), followed by quantitation and statistical analysis. Scale bar, 25 µm. Statistics: ***$p < 0.001$; two-way ANOVA with Tukey's post hoc test, $n = 4$ mice per group; data are presented as mean ± SEM. Related to Figure 7.

Suppl. Figure 14. Graphic summary for this study.

This graphic (also used as the graphic abstract of this paper) illustrates the anti-degenerative role of PTMS in the brain, highlighting the contribution from a population of PTMS-containing EVs released by htNSCs. Within these EVs, PTMS can form a complex with a set of sn/snoRNAs, possibly requiring additional yet-to-be-identified RNA-binding proteins, and this complex can be delivered into neurons to protect against neuronal DNA damage. The study demonstrates that these EVs play a critical role in counteracting neurodegeneration, but this function diminishes under conditions of aging and associated diseases, primarily due to the reduction and loss of htNSCs. Therapeutically, supplementing these EVs can help counteract aging-associated neurodegenerative disorders and diseases such as AD. Related to Discussion.

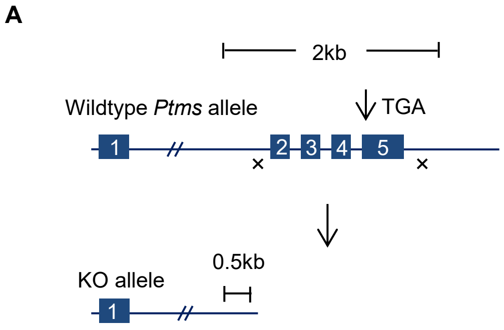
A
2kb
Wildtype *Ptms* allele
TGA
1
2
3
4
5
KO allele
0.5kb
1

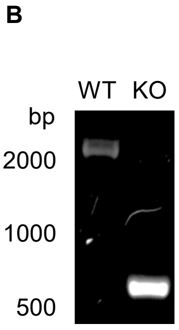
B
WT
KO
bp
2000
1000
500

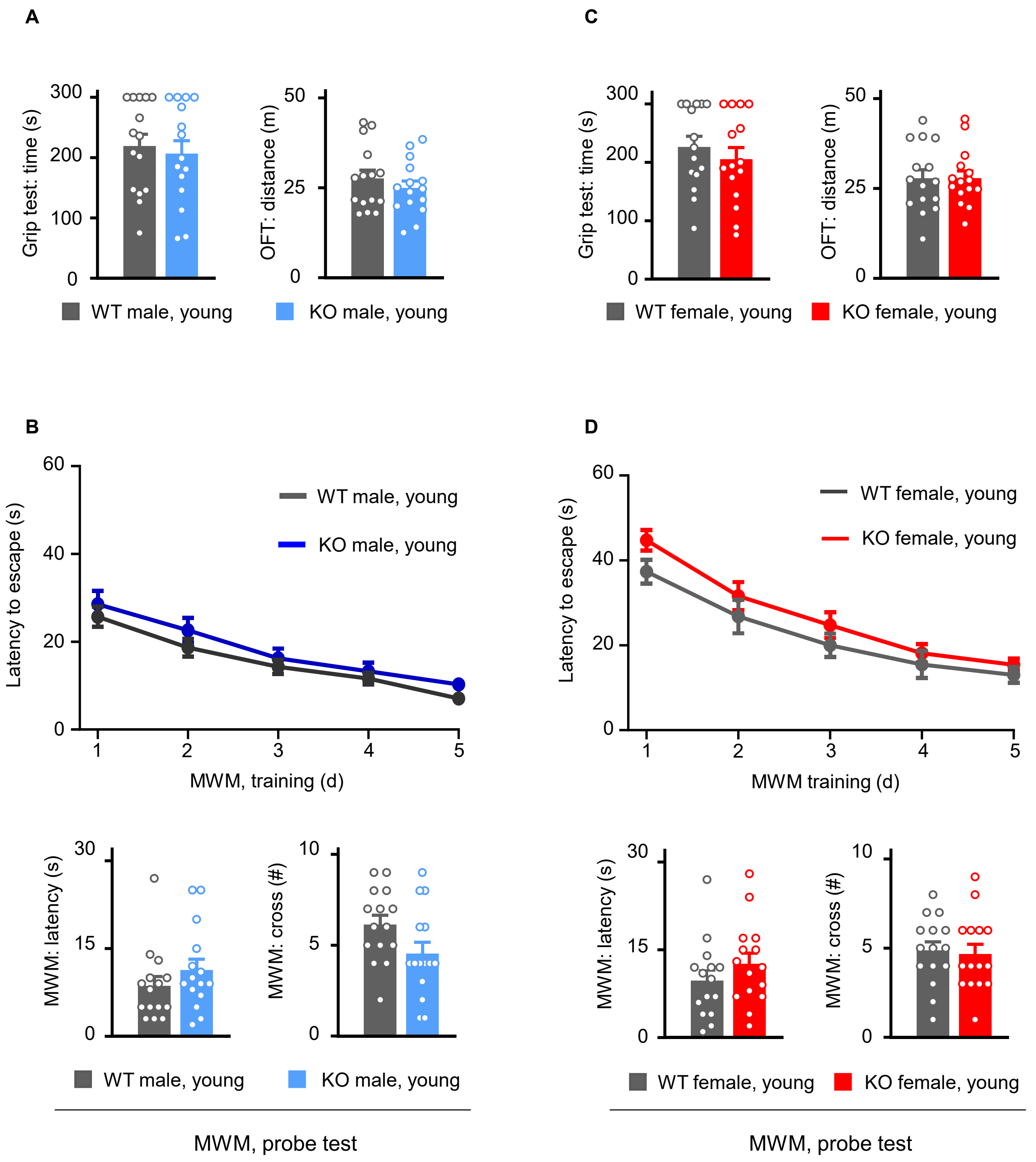
A
Grip test: time (s)
300
200
100
0
OFT: distance (m)
50
25
0
WT male, young
KO male, young
C
Grip test: time (s)
300
200
100
0
OFT: distance (m)
50
25
0
WT female, young
KO female, young
B
Latency to escape (s)
60
40
20
0
WT male, young
KO male, young
1
2
3
4
5
MWM, training (d)
MWM: latency (s)
30
15
0
MWM: cross (#)
10
5
0
WT male, young
KO male, young
MWM, probe test
D
Latency to escape (s)
60
40
20
0
WT female, young
KO female, young
1
2
3
4
5
MWM training (d)
MWM: latency (s)
30
15
0
MWM: cross (#)
10
5
0
WT female, young
KO female, young
MWM, probe test

Suppl. Fig. 3

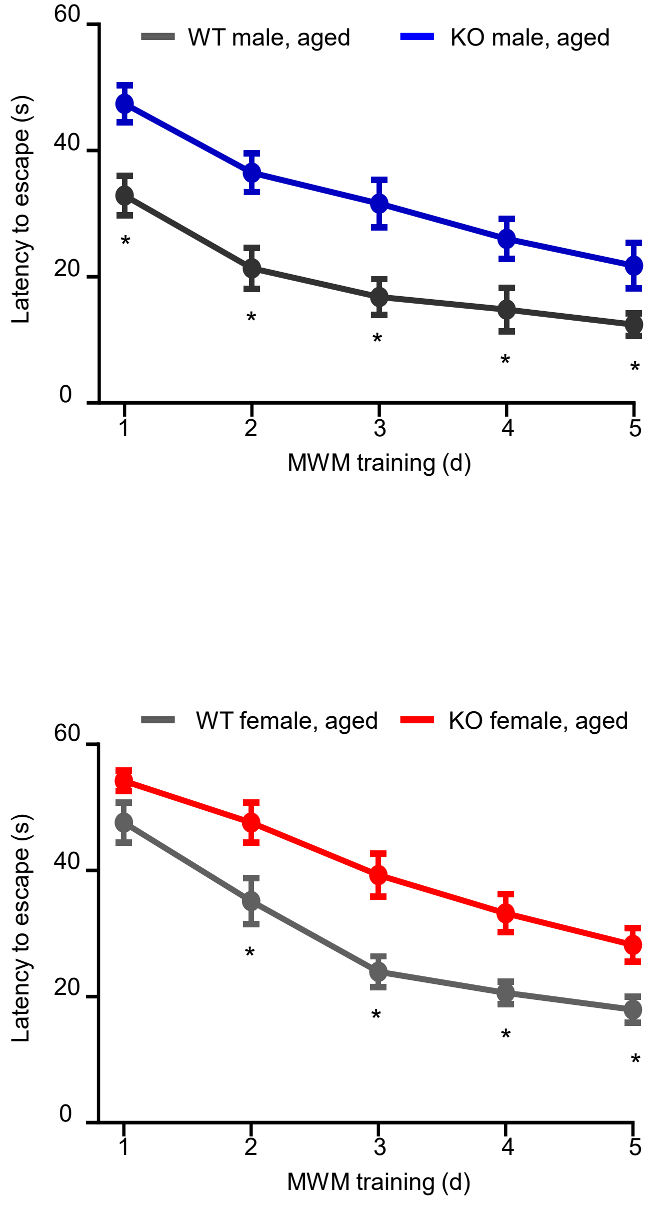

WT male, aged
KO male, aged
Latency to escape (s)
60
40
20
0
*
*
*
*
*
1
2
3
4
5
MWM training (d)
WT female, aged
KO female, aged
Latency to escape (s)
60
40
20
0
*
*
*
*
1
2
3
4
5
MWM training (d)

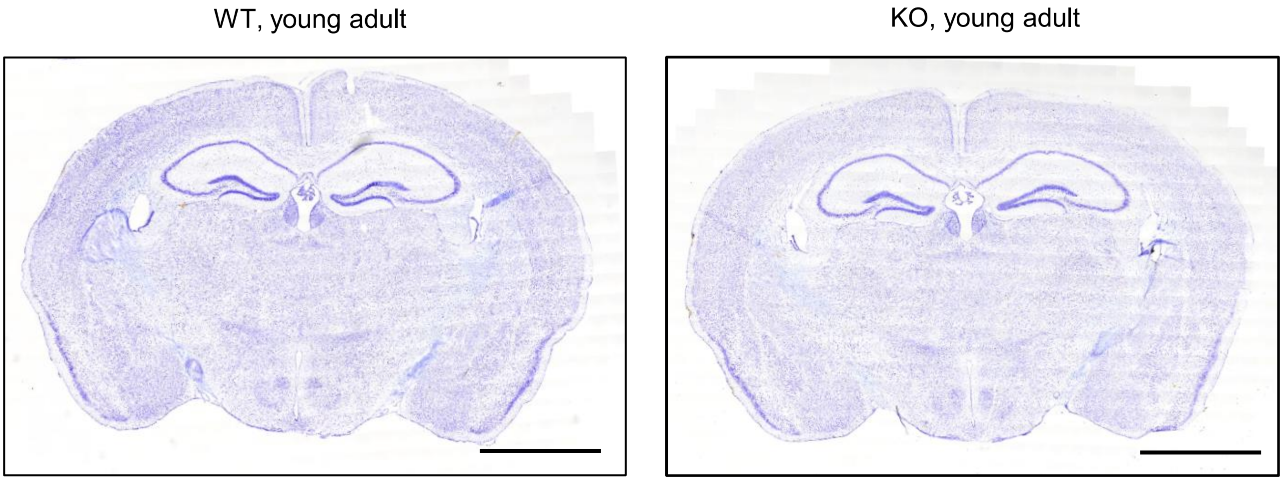
WT, young adult
KO, young adult

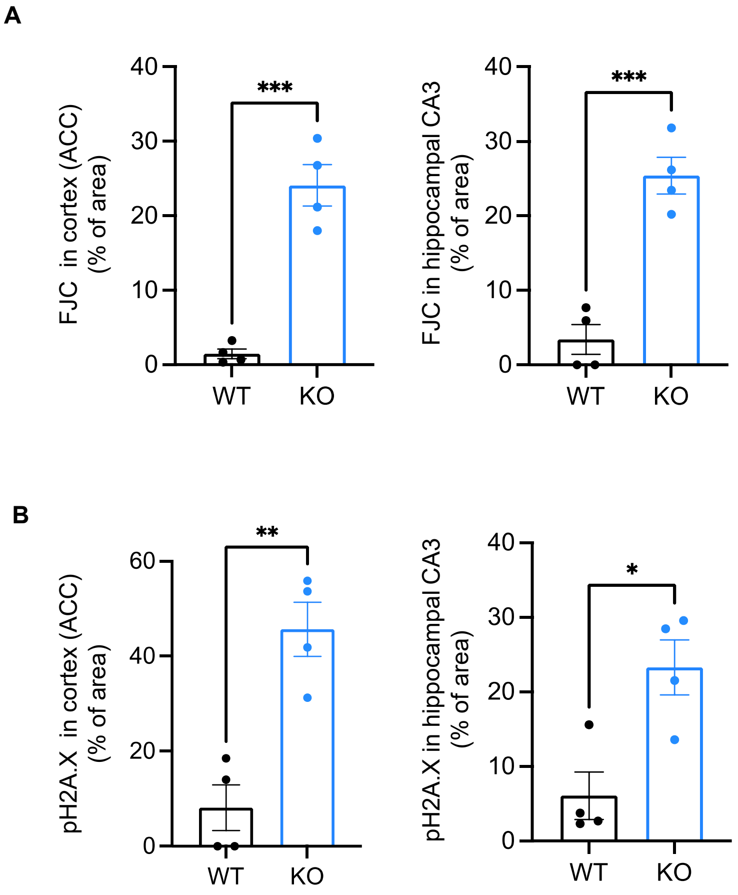
A
FJC in cortex (ACC) (% of area)
FJC in hippocampal CA3 (% of area)
***
***
0
10
20
30
40
WT
KO
B
pH2A.X in cortex (ACC) (% of area)
pH2A.X in hippocampal CA3 (% of area)
**
*
0
20
40
60
WT
KO

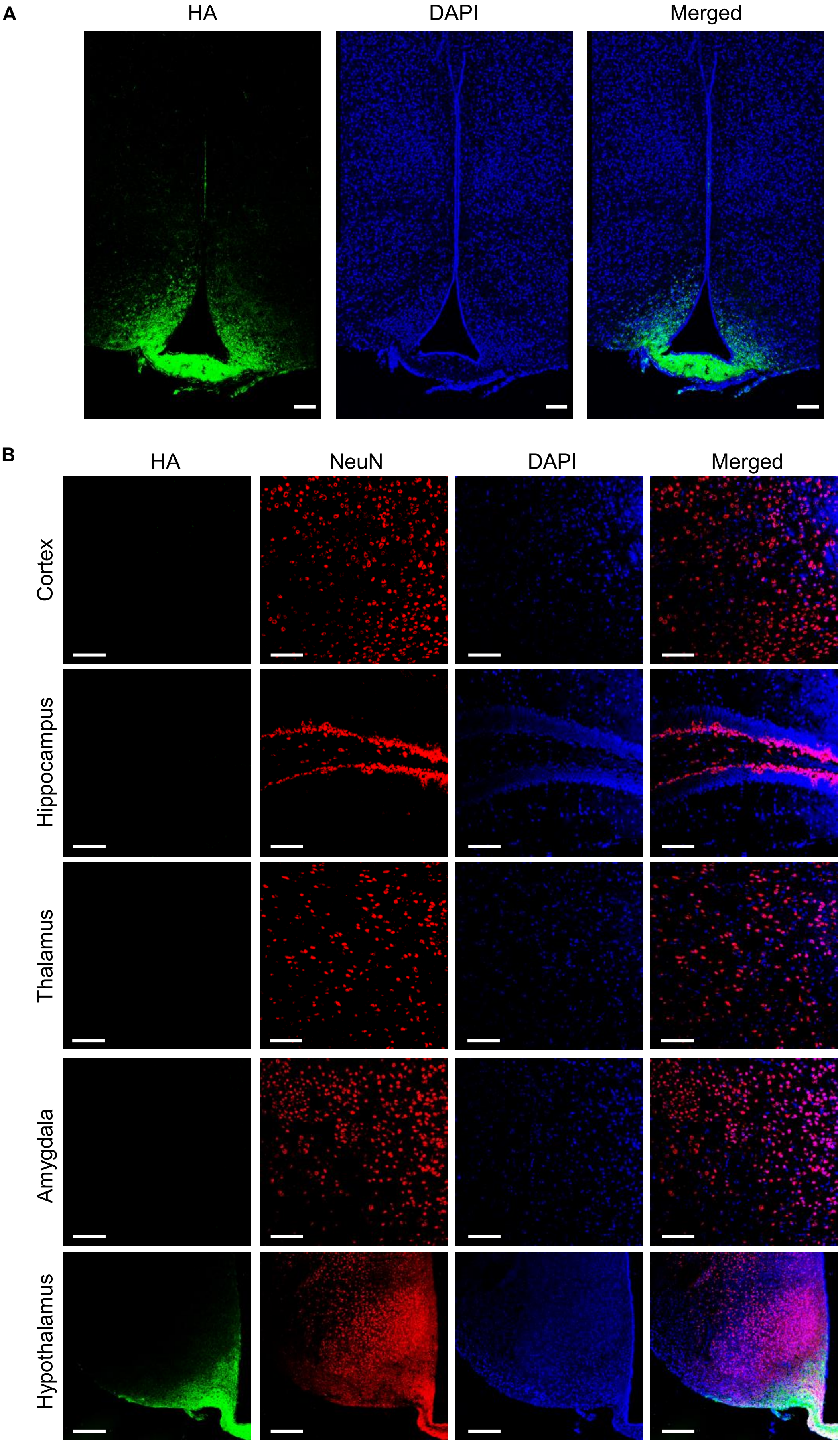

A
HA
DAPI
Merged
B
HA
NeuN
DAPI
Merged
Cortex
Hippocampus
Thalamus
Amygdala
Hypothalamus

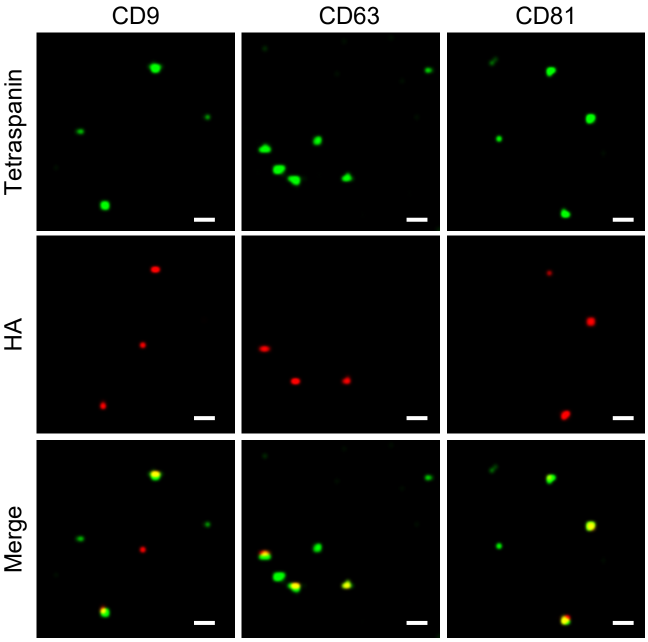
CD9
CD63
CD81
Tetraspanin
HA
Merge

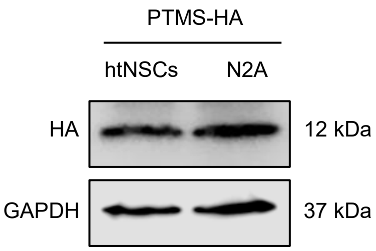
PTMS-HA
htNSCs
N2A
HA
12 kDa
GAPDH
37 kDa

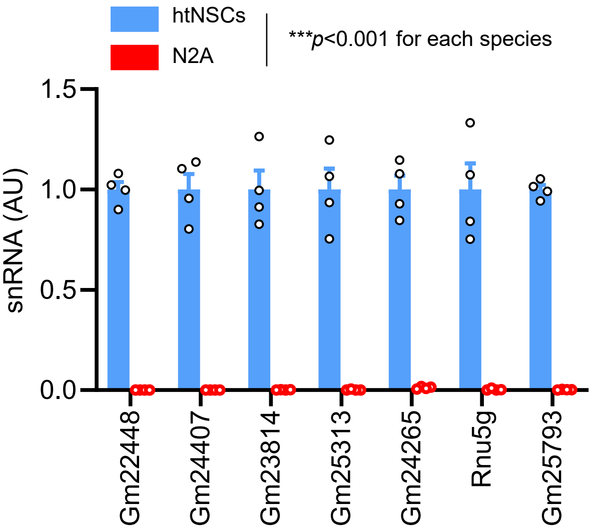
htNSCs
N2A
***p<0.001 for each species
snRNA (AU)
1.5
1.0
0.5
0.0
Gm22448
Gm24407
Gm23814
Gm25313
Gm24265
Rnu5g
Gm25793

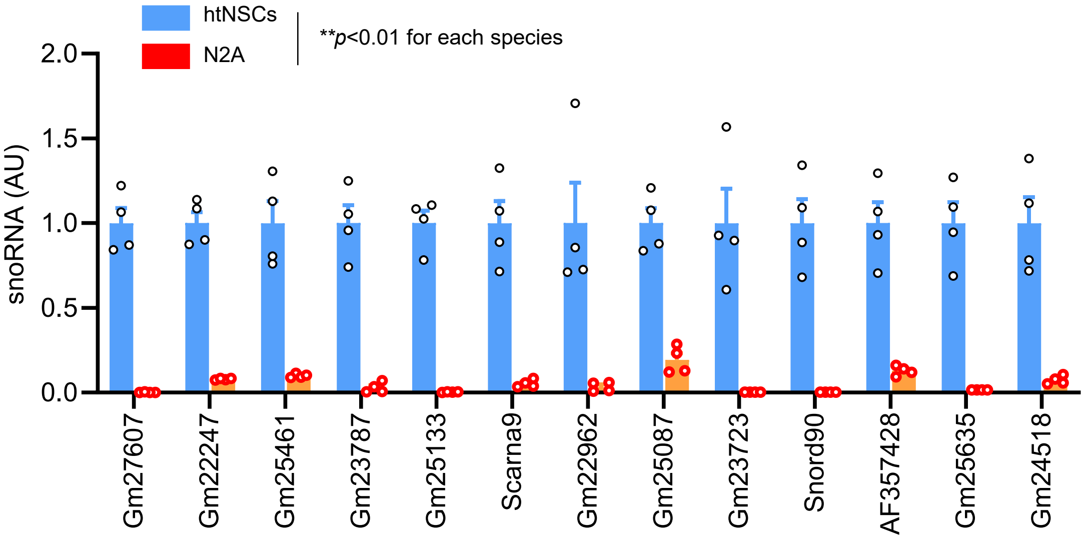
htNSCs
N2A
**p<0.01 for each species
snoRNA (AU)
2.0
1.5
1.0
0.5
0.0
Gm27607
Gm22247
Gm25461
Gm23787
Gm25133
Scarna9
Gm22962
Gm25087
Gm23723
Snord90
AF357428
Gm25635
Gm24518

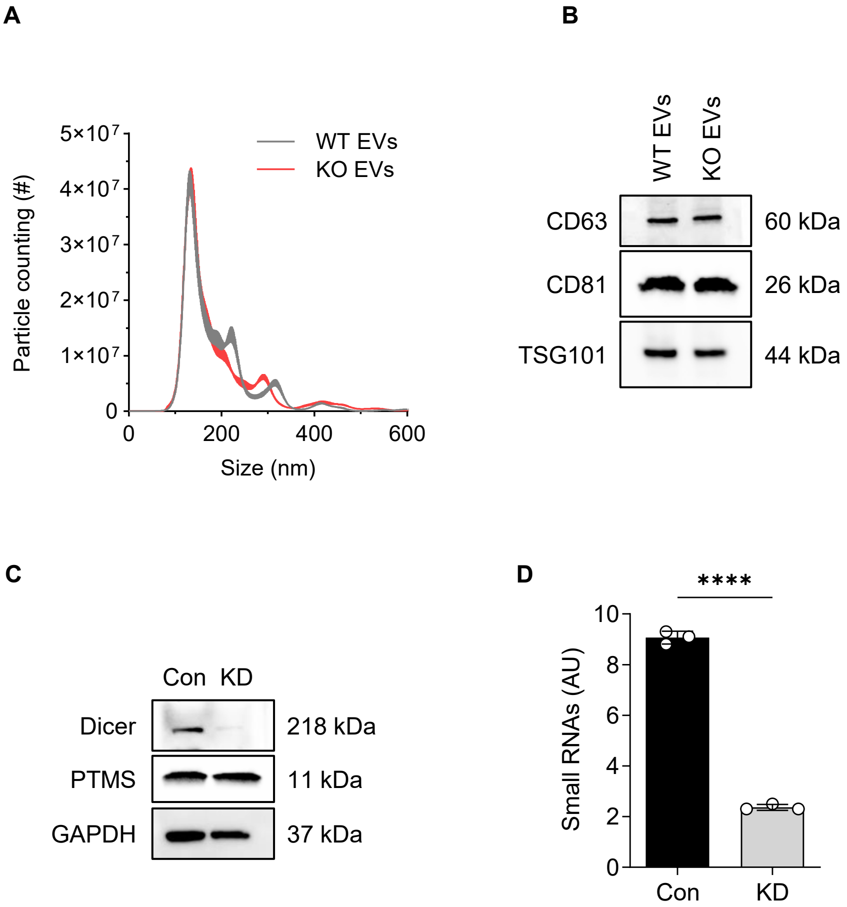
A
5×10^7
4×10^7
3×10^7
2×10^7
1×10^7
0
Particle counting (#)
0
200
400
600
Size (nm)
WT EVs
KO EVs
B
WT EVs
KO EVs
CD63
60 kDa
CD81
26 kDa
TSG101
44 kDa
C
Con KD
Dicer
218 kDa
PTMS
11 kDa
GAPDH
37 kDa
D
****
10
8
6
4
2
0
Small RNAs (AU)
Con
KD

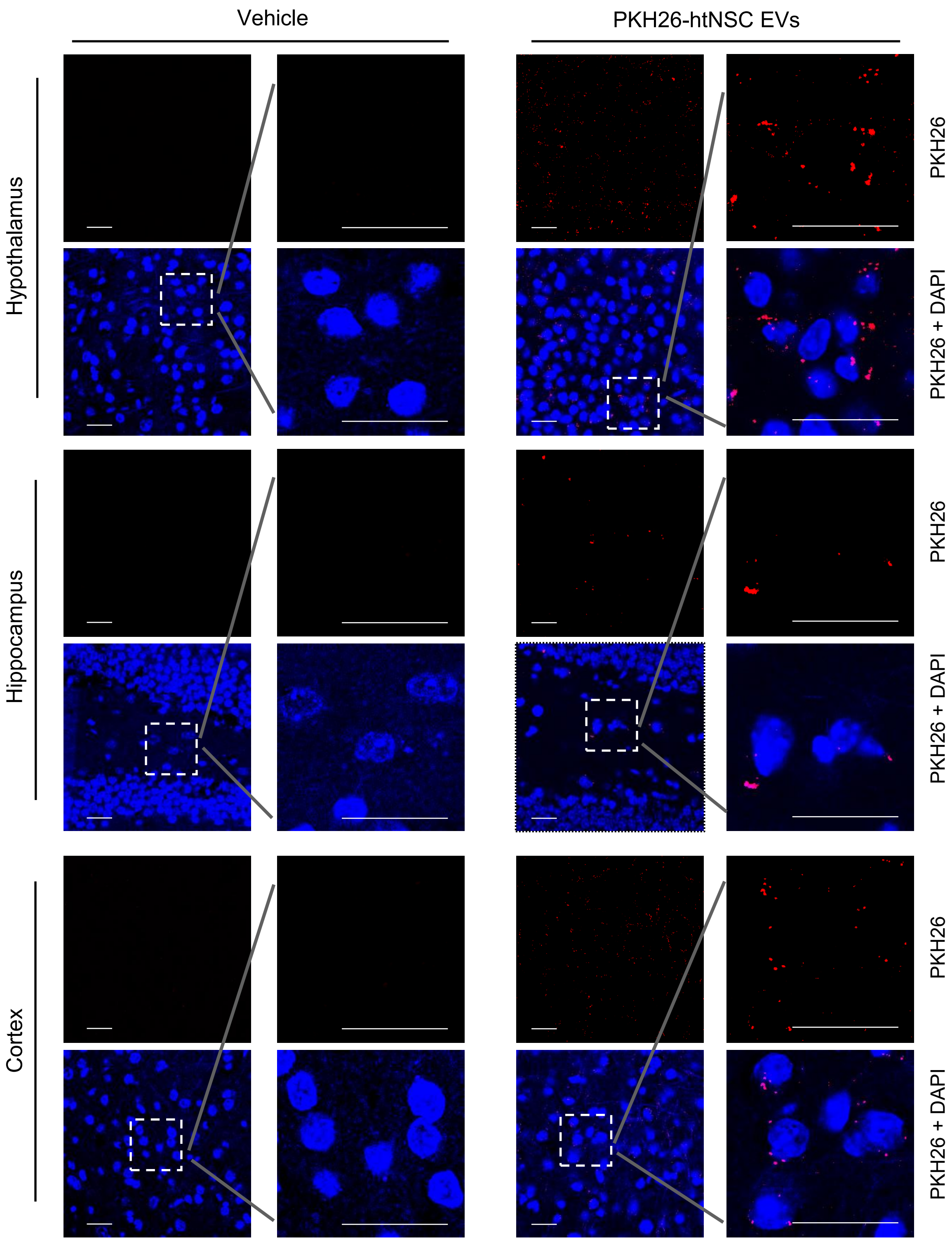

Dash-outlined areas cropped and enlarged for details shown on the right panels

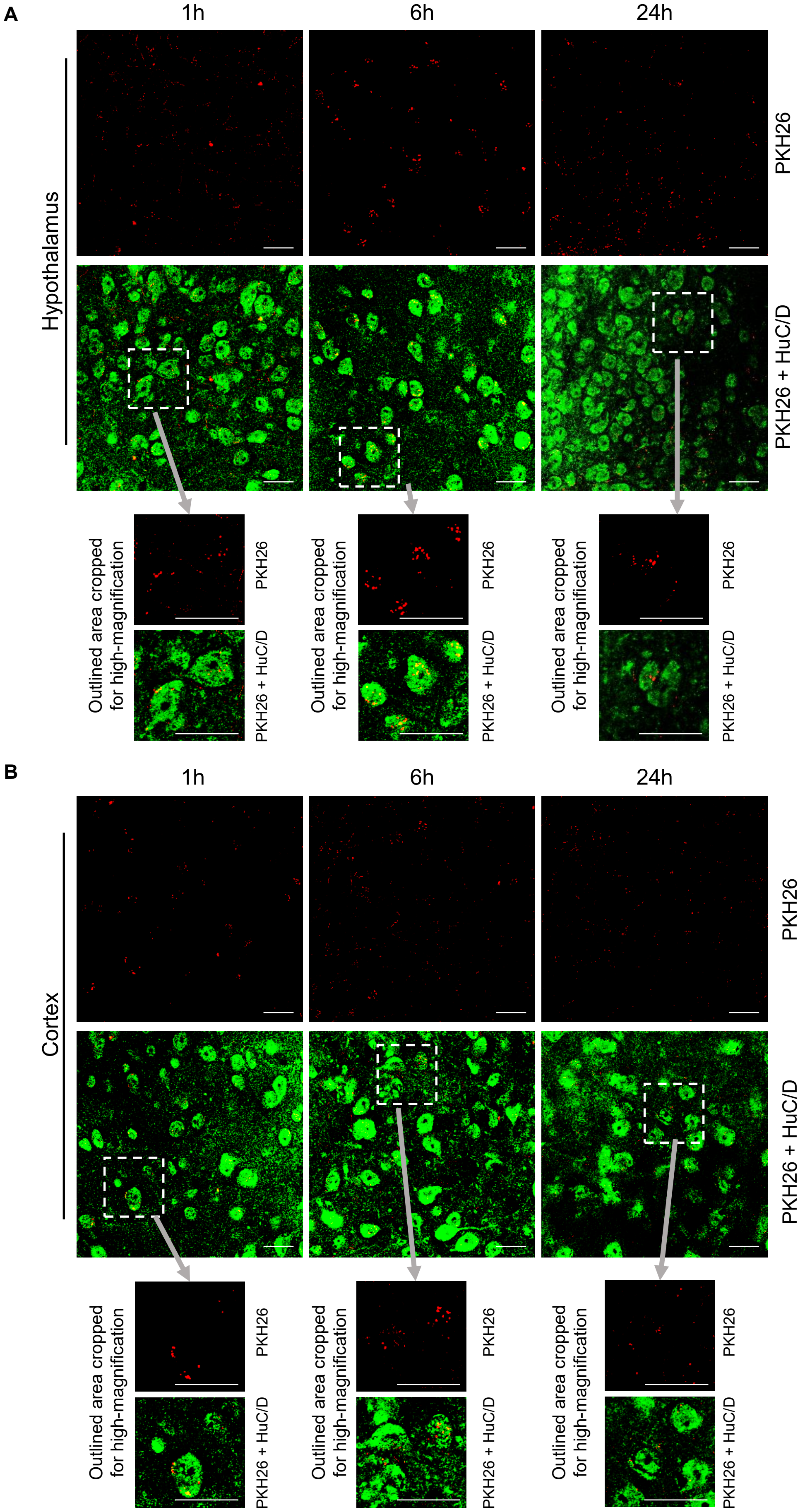
A
1h
6h
24h
Hypothalamus
PKH26
PKH26 + HuC/D
Outlined area cropped for high-magnification
B
Cortex

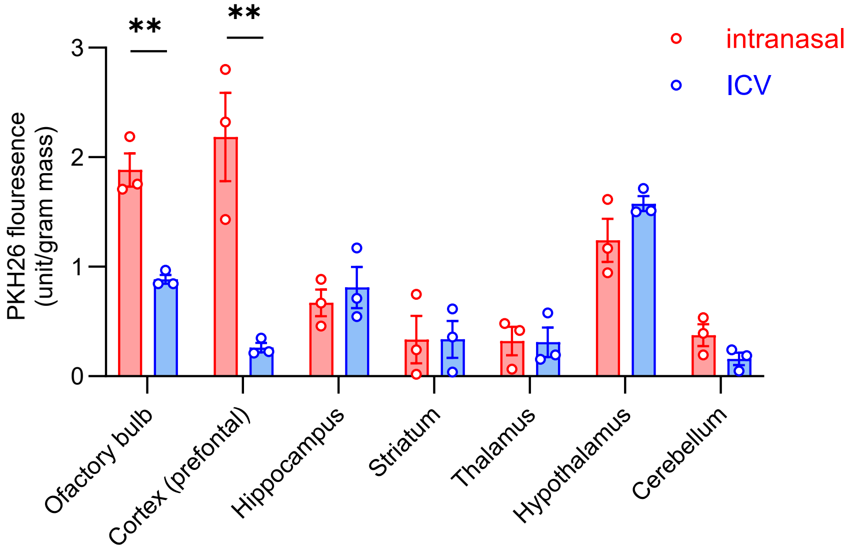
**
**
intranasal
ICV
PKH26 flouresence
(unit/gram mass)
3
2
1
0
Ofactory bulb
Cortex (prefontal)
Hippocampus
Striatum
Thalamus
Hypothalamus
Cerebellum

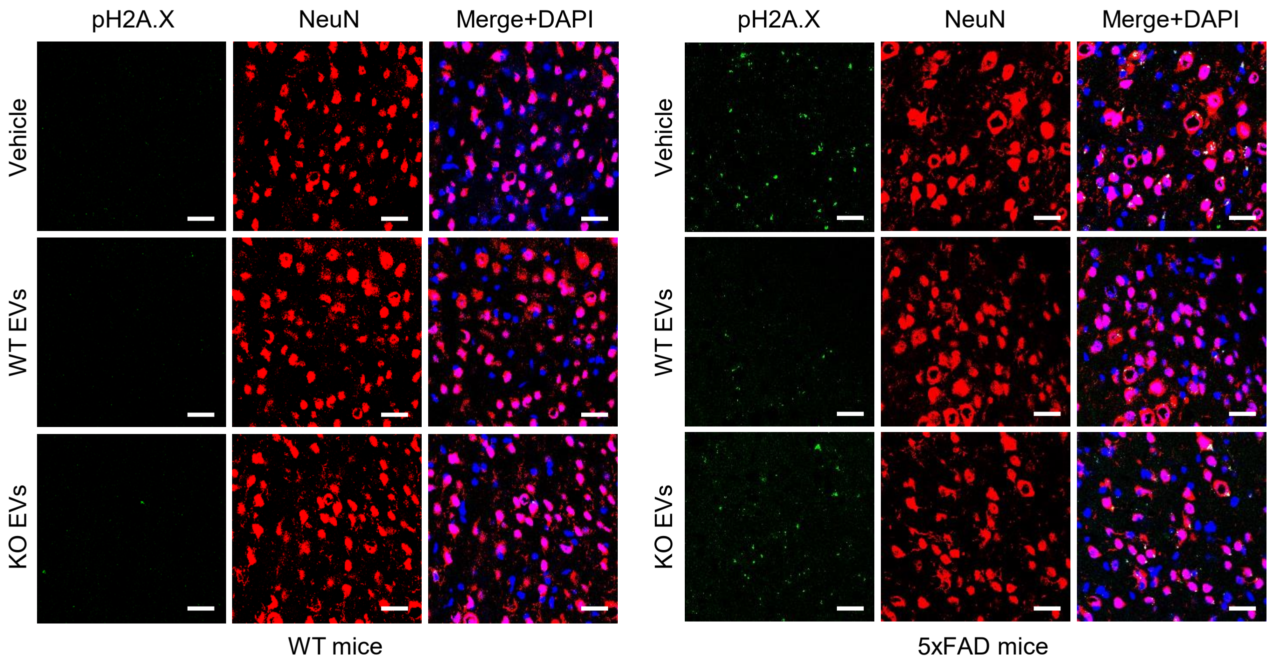
pH2A.X
NeuN
Merge+DAPI
pH2A.X
NeuN
Merge+DAPI
Vehicle
WT EVs
KO EVs
Vehicle
WT EVs
KO EVs
WT mice
5xFAD mice

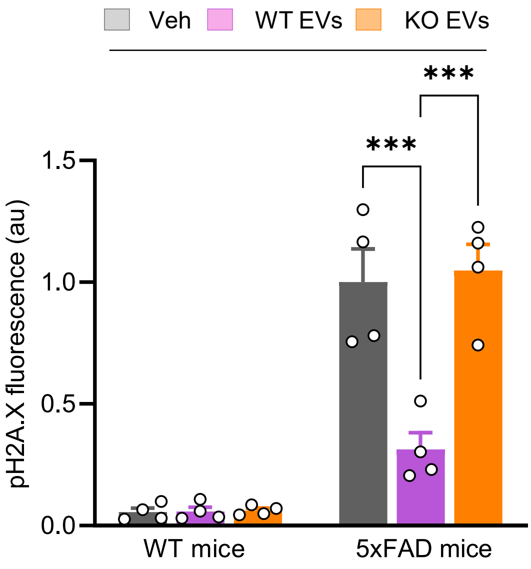
Veh
WT EVs
KO EVs
***
***
pH2A.X fluorescence (au)
1.5
1.0
0.5
0.0
WT mice
5xFAD mice

Suppl. Fig. 14

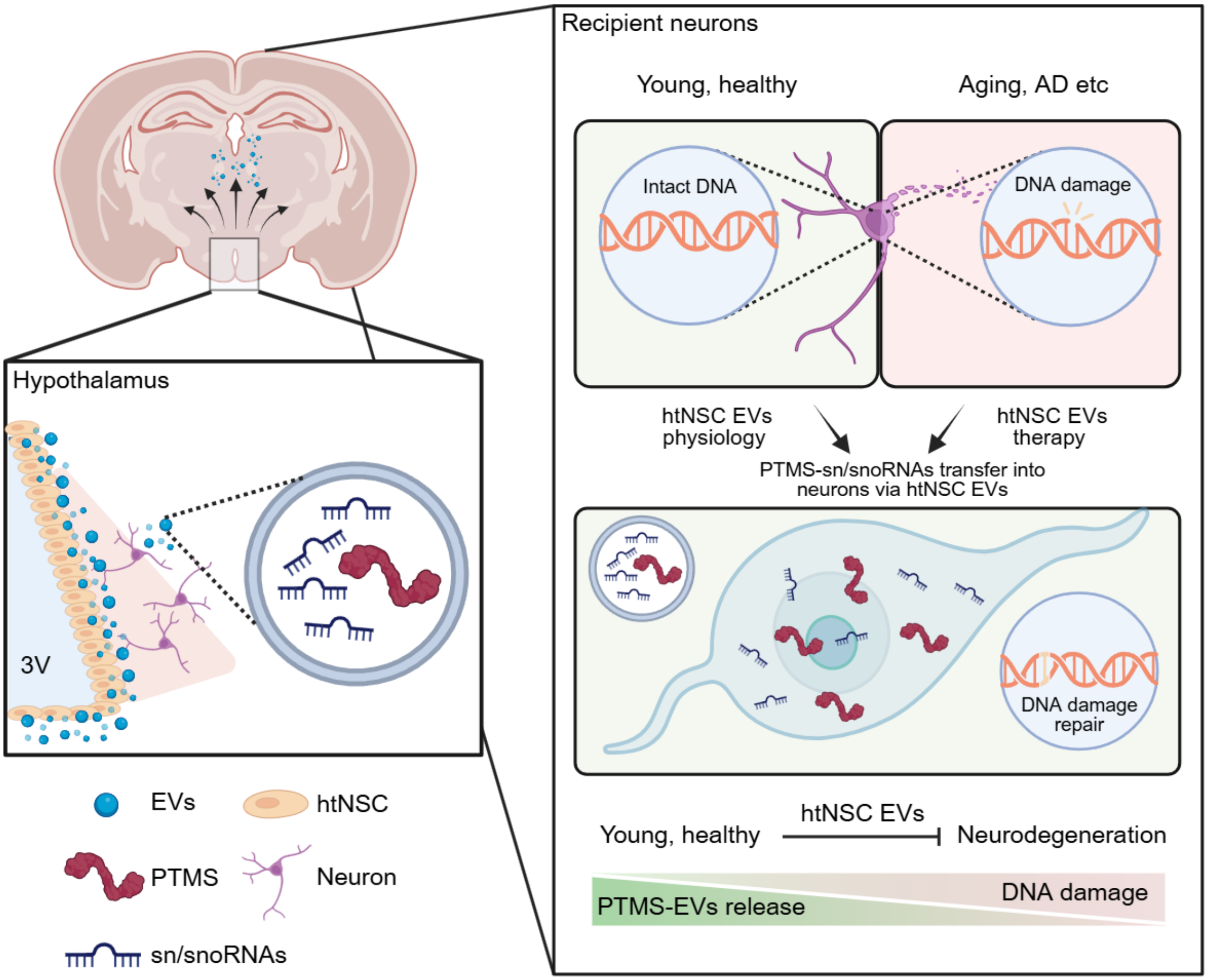
Recipient neurons
Young, healthy
Aging, AD etc
Intact DNA
DNA damage
htNSC EVs physiology
htNSC EVs therapy
PTMS-sn/snoRNAs transfer into neurons via htNSC EVs
DNA damage repair
Young, healthy
htNSC EVs
Neurodegeneration
PTMS-EVs release
DNA damage
Hypothalamus
3V
EVs
htNSC
PTMS
Neuron
sn/snoRNAs
